# The genetic basis of adaptive evolution in parasitic environment from the *Angiostrongylus cantonensis* genome

**DOI:** 10.1371/journal.pntd.0007846

**Published:** 2019-11-21

**Authors:** Lian Xu, Meng Xu, Xi Sun, Junyang Xu, Xin Zeng, Dai Shan, Dongjuan Yuan, Ping He, Weiming He, Yulan Yang, Shiqi Luo, Jie Wei, Xiaoying Wu, Zhen Liu, Xiaomin Xu, Zhensheng Dong, Langui Song, Beibei Zhang, Zilong Yu, Lifu Wang, Chi Zhang, Xiaodong Fang, Qiang Gao, Zhiyue Lv, Zhongdao Wu

**Affiliations:** 1 Department of Parasitology, Zhongshan School of Medicine, Sun Yat-sen University, Guangzhou, China; 2 Key Laboratory for Tropical Diseases Control of the National Ministry of Education, Sun Yat-sen University, Guangzhou, China; 3 Provincial Engineering Technology Research Center for Disease and Vector Control, Guangdong, Guangzhou, China; 4 BGI Genomics, BGI-Shenzhen, Shenzhen, China; University of Texas Health Science Center, UNITED STATES

## Abstract

*Angiostrongylus cantonensis* (rat lungworm) is the etiological agent of angiostrongyliasis, mainly causing eosinophilic meningitis or meningoencephalitis in human. Although the biology of *A*. *cantonensis* is relatively well known, little is understood about the mechanisms of the parasite’s development and survival in definitive hosts, or its adaptation to a broad range of snail intermediate hosts. Here, we generate a high-quality assembly of a well-defined laboratory strain of *A*. *cantonensis* from Guangzhou, China, by using Illumina and PacBio sequencing technologies. We undertake comparative analyses with representative helminth genomes and explore transcriptomic data throughout key developmental life-cycles of the parasite. We find that part of retrotransposons and gene families undergo multiple waves of expansions. These include extracellular superoxide dismutase (EC-SOD) and astacin-like proteases which are considered to be associated with invasion and survival of the parasite. Furthermore, these paralogs from different sub-clades based on phylogeny, have different expression patterns in the molluscan and rodent stages, suggesting divergent functions under the different parasitic environment. We also find five candidate convergent signatures in the EC-SOD proteins from flukes and one sub-clade of *A*. *cantonensis*. Additionally, genes encoding proteolytic enzymes, involved in host hemoglobin digestion, exhibit expansion in *A*. *cantonensis* as well as two other blood-feeding nematodes. Overall, we find several potential adaptive evolutionary signatures in *A*. *cantonensis*, and also in some other helminths with similar traits. The genome and transcriptomes provide a useful resource for detailed studies of *A*. *cantonensis*-host adaptation and an in-depth understanding of the global-spread of angiostrongyliasis.

## Author summary

*Angiostrongylus cantonensis*, rat lungworm, is a common pathogen that causes human eosinophilic meningitis via eating contaminated food. Human angiostrongyliasis has been reported globally. This worm has a complex life-cycle, which includes an especially wide range of snails as intermediate hosts, making it more difficult to eradicate. In this study, we sequenced the genome and transcriptome, and performed comparative analyses to study the potential genetics of its biology using short-read and long-read sequencing technologies. We revealed some potential adaptive evolution in the genome, such as the expansion of retrotransposons and gene families encoding antioxidant enzymes, invasion, migration and digestion related proteases. Specifically, we found a potential clue suggesting convergent evolution of EC-SODs in *Angiostrongylus* and flukes, all of which require snails as intermediate hosts. These results provide an abundant data resource to study the biology and evolution of *A*. *cantonensis* and showed some potential targets against *A*. *cantonensis* and helminths with similar traits.

## Introduction

*Angiostrongylus cantonensis* (rat lungworm) is a parasitic roundworm (nematode) of the superfamily Metastrongyloidea, with a complicated life cycle via a gastropod intermediate host [1]. More than twenty species of *Angiostrongylus* have been discovered in rodents, carnivores and insectivores, and two of them *A*. *cantonensis* and *A*. *costaricensis* are human parasites [1]. *A*. *cantonensis* is the most common infectious cause of eosinophilic meningitis in humans, causing central nervous system (CNS) angiostrongyliasis [2]. Since the first human CNS angiostrongyliasis case reported in 1945 [3], other clinical symptoms including ocular disease, encephalitis and fever of unknown origin have been reported for this disease [4–6]. While most cases were reported in Asia, the Pacific Basin and Australia, human angiostrongyliasis has been found emerging worldwide in the past decades, including USA, France and the UK [7–10] (Figure S1 in S1 Supporting Information).

**Fig 1:**
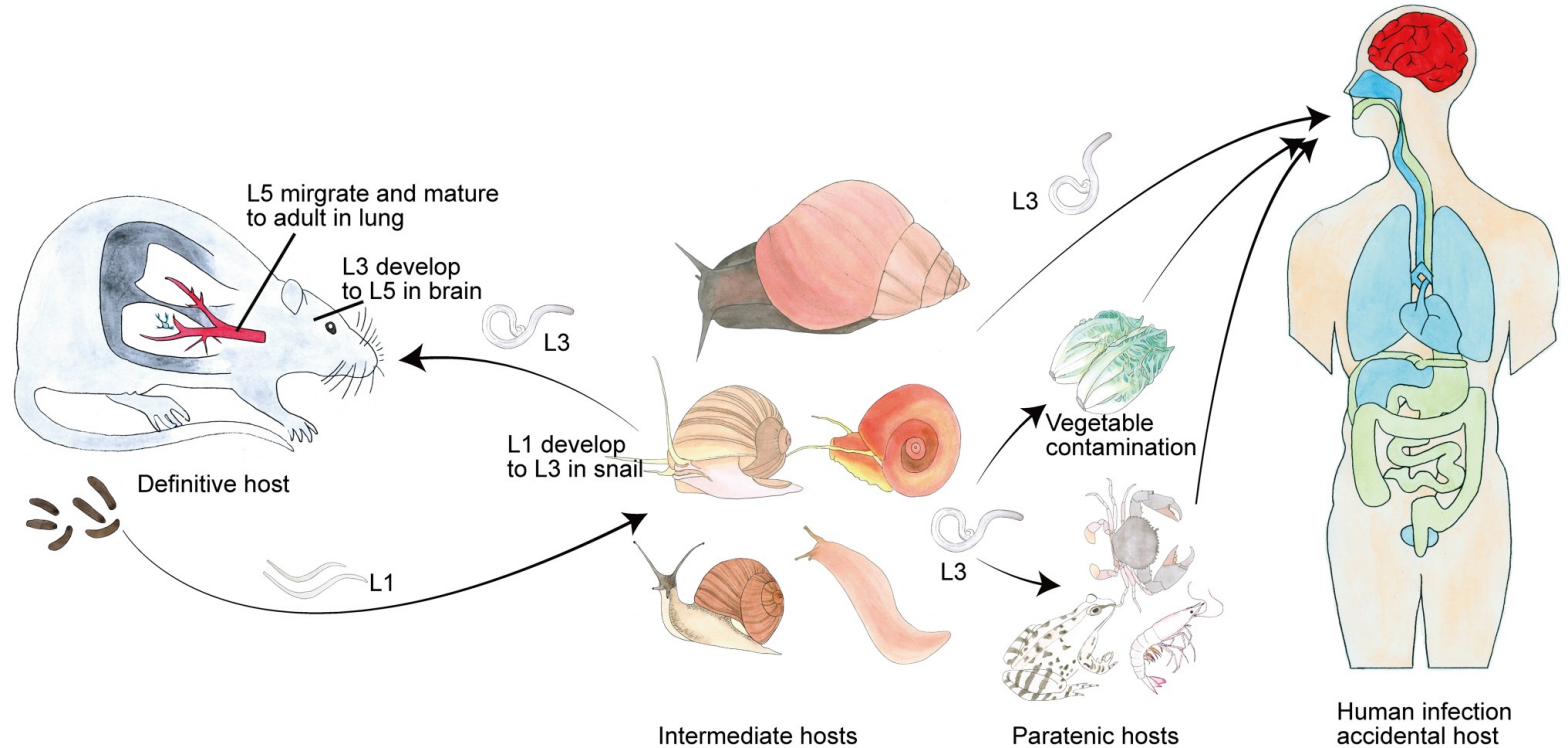
The complex life cycle of *A*. *cantonensis*. The complete life cycle of *A*. *cantonensis* requires two different hosts (snail and rat): L1 larvae are excreted in the feces of a definitive host (rat). When ingested by an intermediate host, they develop into infective L3 after molting twice and are maintained at that stage until they are eaten by a definitive host. The L3 are ingested by a rat and invade intestinal tissue and then migrate to the central nervous system (CNS), where they molt twice and develop into L5. Finally, these worms leave the brain and then reach the pulmonary arteries, where they become fully mature adults. Human infections are acquired by eating undercooked snails, paratenic hosts such as frogs, or contaminated vegetables containing L3 of *A*. *cantonensis*. Since humans are non-permissive hosts of *A*. *cantonensis*, the larvae reach to the brain and cause eosinophilic meningitis.

The life cycle of *A*. *cantonensis* involves a molluscan intermediate host (various species) and a definitive rodent host (*cf*. review [11], Fig 1). Briefly, the first-stage larvae (L1) are swallowed by an intermediate host, they molt twice into third-stage larvae (L3). The infective L3 are ingested by a definitive host, then they migrate to the brain and molt twice into young adults (L5). Eventually, the L5 migrate to the lungs where develop to sexual maturity and lay eggs. The eggs embryonate, develop and hatch to L1 and they are excreted in host feces, restarting the life cycle. This worm can infect a very wide range of intermediate hosts, comprising at least 160 species belonging to 44 families of freshwater and land gastropods [12]. The two available assemblies are highly fragmented in nature which has posed as an obstacle to detailed biological and evolutionary investigations [13, 14].

In the present study, we sequenced and assembled a high-quality reference genome of a well-defined laboratory strain of *A*. *cantonenis* from Guangzhou, China. Through analyses of comparative genomics and transcriptome, we explored potential molecules regarding the nematode survival in intermediate host and/or definitive host.

## Methods

### Ethics statement

Procedures involving animals and their care described here were approved by the Institutional Animal Care and Use Committee of Sun Yat-sen University (Permit No: 2016–055) and followed the National Guidelines for Experimental Animal Welfare (MOST, China, 2006).

### Sample preparation and sequencing

The life-cycle of *A*. *cantonensis* was established and maintained in the Department of Parasitology, Zhongshan School of Medicine. L1 larvae were separated from feces of rats. L3 were isolated from experimentally infected snails using the method previously described by Zeng [15]. Sprague Dawley rats were challenged with L3 (200 per animal) via intragastric administration. Procedures for animal care described herein were approved by the Institutional Animal Care and Use Committee of Sun Yat-sen University. Other developmental worms (L4, L5 and mature adults, including female and male) were harvested from rats at 21, 28, and 48 days post-infection (dpi) respectively. Genomic DNA was extracted from ten adult worms. Seven paired-end and mate-pair whole-genome shotgun libraries (250bp, 500bp, 800bp, 2kb, 5kb, 10kb, and 20kb, Table S1 in S2 Supporting Information) were constructed and then sequenced using the Illumina HiSeq 2000 platform. Another 20 kb library for PacBio sequencing was constructed and sequenced using RSII. RNA was extracted from different developmental stages of *A*. *cantonensis* (L1, L3, L4, L5 and mature adults, the *Pomacea canaliculata* used as an intermediate host), respectively. Seven cDNA libraries were sequenced using the Illumina Hiseq 2000 platform. Another four cDNA libraries (L3 and L4, the *Biomphalaria glabrata* used as an intermediate host) were constructed and sequenced using the Illumina HiSeq 4000 platform.

### Genome assembly and annotation

We employed a hybrid assembly strategy by combining Illumina and PacBio data (Figure S3 in S1 Supporting Information). Illumina reads were first assembled into contigs using the Platanus [16] (v1.2.4) with default parameters. The resulting Illumina contigs and PacBio subreads were further used to assemble with DBG2OLC [17] pipeline (release Jun 2015). Then, correction of the assembly was performed twice with Pilon [18] (v1.22) by using Illumina reads. To further link the corrected contigs, the corrected PacBio reads and Illumina mate-pair reads were employed to extend and link into scaffolds using SSPACE-LongRead [19] (v1–1) and SSPACE [20] (v2.0). Remaining gaps within these scaffolds were filled with GapCloser available in SOAPdenovo2 [21]. CEGMA (Core Eukaryotic Genes Mapping Approach) [22] (v2.4), BUSCO (benchmarking universal single-copy orthologues) [23] (v3.0.1), and *de novo* assembled transcripts with Trinity [24] (v2.0.6) were used to assess the completeness of the assembly.

Protein-coding gene models were predicted using a strategy to combine the homology-based prediction and RNA-seq data as previously described [25] (*cf*. S1 Supporting Information, Supplementary Methods and Results sections). The functional annotation of protein-coding genes was performed using BLASTP alignment to databases: Swiss-Prot (release Jun 2019), TrEMBL (release Jun 2019), NCBI NR (release Sep 2017) and KEGG (release 89). InterPro domains and GO terms were assigned with InterProScan [26] (release 5.3).

Repetitive elements (REs) in the assembly were identified using a combination of homology- and *ab initio*-based approaches. RepeatMasker and RepeatProteinMask (http://www.repeatmasker.org/, version open-4-0-5) were applied to detect homologous REs in the RepBase database (v20.04). PILER [27] (v1.0), RepeatScout [28] (v1.0.5), and LTR-Finder [29] (v1.0.6) were used to build a *de novo* repeat library. RepeatMasker was run against the *de novo* library. The same pipeline was employed to predict REs in seven other nematode genomes (*Ascaris suum* [30], *Brugia malayi* [31]; *Caenorhabditis elegans* [32], *Haemonchus contortus* [33, 34], *Necator americanus* [35], *Meloidogyne hapla* [36]). For RTE-RTE transposable elements, proteins of RTE-RTEs deposited in RepeatPep (RepeatMasker-open-4.0.6) were collected and used to search in eight nematode genomes using homology-based prediction pipeline as delineated in gene prediction, except with an alignment rate of more than 50%. Amino acid sequences encoding a reverse transcriptase (RT, PF00078) domain were aligned using MUSCLE [37] (v3.8.31) and then were constructed a phylogenetic tree using FastTree [38] (v2.1).

### Genome evolution

The OrthoMCL [39] pipeline was used to determine orthologous groups in *A*. *cantonensis* and seven other represented nematode genomes (*A*. *suum*, *B*. *malayi*; *C*. *elegans*, *H*. *contortus*, *N*. *americanus*, *M*. *hapla* and *T*. *spiralis*, related data was downloaded from the WormBase [40] (version 246). 788 single-copy orthologous genes were extracted to build a phylogenetic tree. Sequences from each single copy orthologs were aligned using MUSCLE and then filtered with trimAl [41] (v1.2) with default parameters except “-gt 0.5”. RAxML [42] (v8.2) was used to construct gene tree with “GTRGAMMA” model. Finally, ASTRAL [43] (v5.6.1) was used to construct species tree based on 788 gene trees. Phylogenetic relationship among eight nematodes and six flatworms (*Schistosoma japonicum* [44], *S*. *mansoni* [45], *S*. *haematobium* [46], *Opisthorchis viverrini* [47], *Clonorchis sinensis* [48] and *Schmidtea mediterranea* [49], related data was downloaded from WormBase Parasite [50], WBPS5) was resolved using the method described above based on 173 single-copy orthologous genes. Species divergence time was estimated using MCMCTREE, which is part of the PAML package [51] (v4.5). Published times for *T*. *spiralis* and *C*. *elegans* (~428 million years ago, mya), and *B*. *malayi* and *C*. *elegans* (~241 mya) divergence were used to calibrate divergence time [52]. We investigated the expansion and contraction of gene families using the CAFÉ [53] (Computational Analysis of gene Family Evolution, v2.1), which infers the dynamics of the gene family under a stochastic birth and death model.

### Identification, evolution and expression of specific gene or gene families

To avoid systematic biases, for example, different methods in the annotation of the previously published helminth draft genomes, we adopted a uniform strategy to re-annotate and check candidate genes screened from the above comparative analysis. Generally, protein sequences of superoxide dismutase (SOD) genes, astacin-like genes, and several hemoglobin digestion proteases of nematodes deposited in Swiss-Prot or MEROPS [54] (download in Nov 2016) databases were downloaded and mapped to the genomes using the homology-based gene prediction. We also manually checked these putative genes and compared with the original gene annotation (The associations of the gene IDs used in this study and the gene IDs in Wormbase are listed in Table S9 in S2 Supporting Information). Phylogenomic analyses of the gene families studied herein were based on protein sequences. The best model of amino acid replacement was estimated using ProtTest [55] (v3.4.2) software. The phylogenetic trees of these genes were constructed using PhyML [56] (v3.0) software, respectively. For EC-SODs, we also reconstructed the phylogenetic trees using RAxML and IQ-TREE [57] (v1.6.5), and conducted a hypergeometric test site by site at amino acid level to detect the potential convergent evolution [25] between the genus *Angiostrongylus* and flukes in a broad range of 141 EC-SODs from 62 species (43 nematodes and 19 platyhelminths, Table S10 in S2 Supporting Information).

For RNA-seq analysis, we mapped RNA-seq reads to the genome with Tophat2 [58] (v2.0.8). We quantitated the gene expression level using uniquely mapped reads and measure in reads per kilobase per million reads (RPKM). The expression of EC-SOD and MTP-1 subclade I and II genes were validated using real-time PCR (qPCR, primers are shown in Table S8 in S2 Supporting Information), with pooled larvae/adults isolated from multiple hosts for each developmental stage. β-actin was used as an internal control. The relative changes in gene expression were calculated by equation 2^−ΔΔC^_T_, where ^ΔΔC^_T_ = (CT,target—C_T,Actin_)_Time x_—(C_T,Target_—C_T, Actin_)_Time 0_. Time x is any time point and Time 0 represents the 1 × expression of the target gene normalized to β-actin [59].

## Results

### Genome assembly and annotation

The genome of *A*. *cantonensis* was sequenced using the Illumina HiSeq 2000 and PacBio RSII platforms, yielding a total of ~267-fold and ~41-fold coverage, respectively (estimated genome size: 290 Mb; Figure S2 in S1 Supporting Information and Table S1 in S2 Supporting Information). The final genome assembly included 282 Mb in 816 contigs and 425 scaffolds, with a contig N50 of 993 kb and a scaffold N50 of 1.8Mb (Table 1). The assembly covered more than 95% (coverage ≥ 70%) of the assembled RNA-seq transcripts, indicating that the gene region was well represented (Table S2 in S2 Supporting Information). In addition, both the CEGMA and BUSCO methods were used, and the results showed that the assembly in this study was more complete than the published fragment assemblies of *A*. *cantonensis* in the protein-coding region (Table 1). Taken together, the results showed that the present genome assembly of *A*. *cantonensis* represented a substantial part of the whole genome. Combined homology-based and RNA-seq methods, we predicted 13,473 protein-coding gene models (5.16% of the assembly, spanning ~15 Mb) in *A*. *cantonensis* genome. Of these genes, 13,114 (97%) were supported by RNA-seq data (RPKM≥1 at least one sample), and 12,407 (92.1%) genes either had homologues in public databases (Swiss-Prot, KEGG, and NCBI NR).

**Table 1:** Statistics of the *A*. *cantonensis* assemblies.

	This study	Yong *et al* [14].	Avril *et al* [13].
Assembly size (Mb)	283	260	253
Scaffold number^a^	425	16,326	18,635
Gaps (bp)	1,480,142	25,114,564	4,505,497
Contig N50 (kb); Scaffold N50 (kb)	993; 1,815	1.7;42.2	27.1;43.9
GC content %	41.7	41.2	41.5
Complete BUSCOs[Duplicated]^b^	84.3%[1.2%]	62.5%[0.8%]	70.0%[1.2%]
Fragmented BUSCOs	7.80%	12.30%	10.20%
Missing BUSCOs	7.90%	25.20%	19.80%
CEGMA completeness	97.98%	80.24%	82.66%

^a^, length cut-off: 500bp;

^b^, Nematoda_odb9 dataset was used

**Fig 2:**
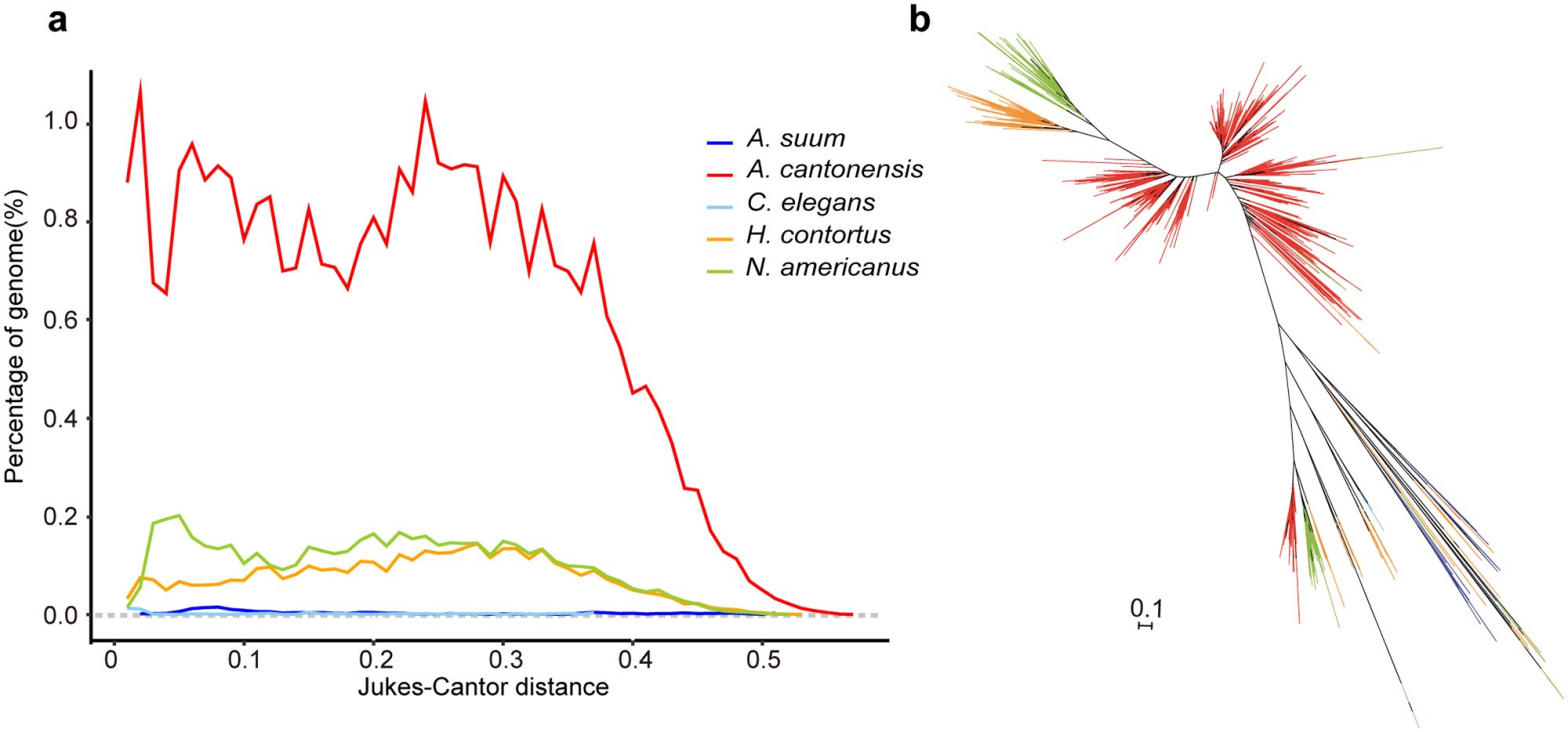
RTE-RTE retrotransposons expansion in *A*. *cantonensis*. **(a)**, The genomic portion of the RTE-RTEs in eight nematodes (three of which lack RTE-RTEs using the same criterion) at different given divergence generated by the *de novo* prediction. The divergence is adjusted for multiple substitutions using the Jukes-Cantor distance. **(b),** The phylogenetic tree depicting the relationship of RTE-RTEs among five nematodes. The branches of five species of nematodes are colored in blue for *A*.*suum*, red for *A*. *cantonensis*, light blue for *C*. *elegans*, orange for *H*.*contorus* and green for *N*. *americanus*.

Transposable elements (TEs) represented 54.61% of the assembled genome, representing a greater percentage than most parasitic nematode genomes characterized to date (3.4–32.9% using the same bioinformatics pipeline; Table S3 in S2 Supporting Information). RTE-RTE retrotransposons, belonging to the long interspersed elements (LINEs), were the most abundant group in *A*. *cantonensis* genome, representing 39.2% of the genome and 72% of repeats respectively (Table S4 in S2 Supporting Information), a markedly higher percentage than the genomes of other studied nematodes (0~5.1%; Table S3 in S2 Supporting Information). Sequence divergence of extant RTE-RTE copies compared to the repeat consensus showed at least two periods of element expansion in *A*. *cantonensis* (Fig 2a). The phylogeny showed that the RTE-RTEs expanded independently in *A*. *cantonensis*, *N*. *americanus*, and *H*. *contortus*, but the *A*. *cantonensis* displayed substantially higher divergence and abundance than the other two strongyloids (Fig 2).

### Genome evolution

To better understand the evolution of *A*. *cantonensis* genome and to infer genes or gene families associated with parasitism, we performed a comparative analysis with seven other nematode genomes representing clades I, III, IV and V (*A*. *suum*, *B*. *malayi; C*. *elegans*, *H*. *contortus*, *N*. *americanus*, *M*. *hapla* and *T*. *spiralis*). We identified 788 one-to-one orthologous genes in all eight nematodes and assessed the phylogenetic relationships using a coalescent-based method [43]. The phylogenetic analysis showed that *A*. *cantonensis* was genetically closer to *H*. *contortus* (Figure S5 in S1 Supporting Information), which was the same as the study of the 50 Helminths Genome Project [13]. We inferred that 26 and 119 gene families respectively underwent significant expansion and contraction in the *A*. *cantonensis* lineage (Viterbi *P*≤0.05; Tables S6 and S7 in S2 Supporting Information, Figure S6 in S1 Supporting Information). Expanded genes included protease (neprilysin-1 and legumain), transporter (sodium-dependent high-affinity dicarboxylate transporter 3), receptor (acetylcholine receptor) and ancylostoma secreted protein. Based on the OrthoMCL cluster result, we also identified 454 genes (159 groups; Table S5 in S2 Supporting Information) that appeared to be unique in *A*. *cantonensis*, which were significantly enriched in GO terms of ʺSuperoxide dismutase activityʺ (GO:0004784) and metallopeptidase activityʺ (GO:0008237) (adjusted *P*-value < 0.05, Figure S4 in S1 Supporting Information). These ʺuniqueʺ genes included astacin-like metalloproteinase (M12A), Aspartic protease (A01) and Extracellular superoxide dismutase (EC-SOD) which are likely related to the invasion, migratory and digestive processes, innate immune of *A*. *cantonensis*. Those expanded or specific genes may provide novel clues to *A*. *cantonensis* adaptation to hosts.

### Expansion of EC-SOD genes related to host adaptation in *A*. *cantonensis*

Reactive oxygen species (ROS), such as oxygen radicals and superoxide, are generated by phagocytes (vertebrates) or haemocytes (invertebrates), and represent an innate defense system against pathogens [60–62]. Helminth parasites secrete antioxidant enzymes for defense against host-generated ROS for survival in the host [60]. Extracellular superoxide dismutase (EC-SOD, also known as SOD3) belongs to the SOD gene family, which converts superoxide radical into hydrogen peroxide and represents the first step in the antioxidant enzyme system to reduce ROS [60].

**Fig 3:**
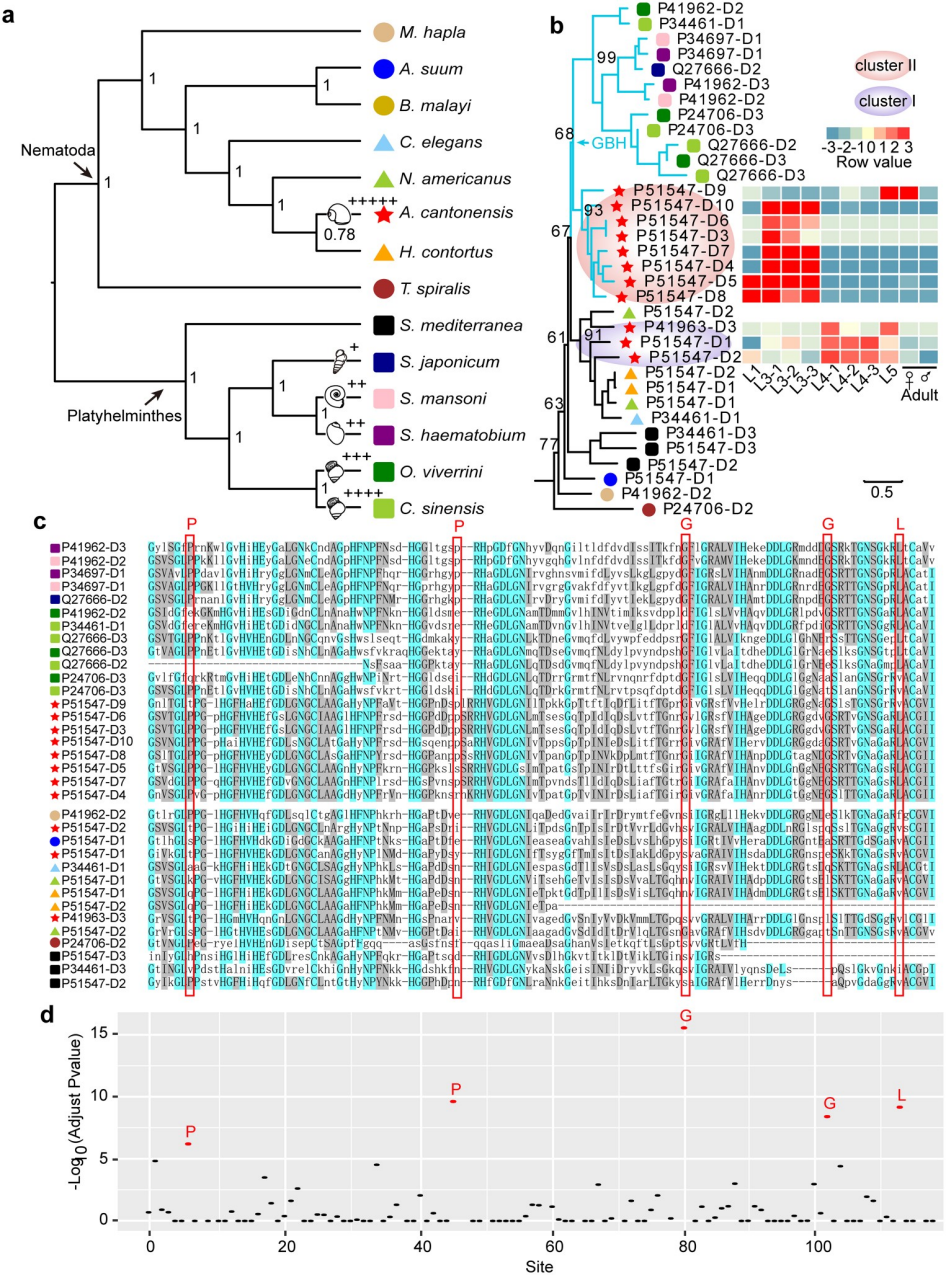
Phylogenomic analysis of EC-SOD in different species of nematodes and flatworms. **(a),** Phylogenic tree depicts a cladogram of eight nematodes and six flatworms profiled in this study. Number at the node indicates ASTRAL supporting value while the branches with the sketch of snails represents intermediate hosts. Specifically, the number of ʺ+ʺ shows the increasing spectrum of suitable intermediate hosts. **(b),** Maximum likelihood tree of EC-SODs in 14 species and the mRNA expression patterns of *A*. *cantonensis’s* EC-SODs. **GBH**: the EC-SOD clustered in gastropod-borne helminths. **(c),** The multiple sequence alignment of EC-SODs from sequences in **Fig 3b**. **(d)**, Convergent study at amino acids levels in the EC-SODs from gastropod-borne helminths at an extended background of 62 species. The x-axis shows multiple sequence alignment position in Fig 3c.

Eleven tandem EC-SOD genes were identified in the *A*. *cantonensis* genome, which were also confirmed by PCR using genomic DNA from *A*. *cantonensis* (Figure S9 in S1 Supporting Information), which was four times more than the number in the other seven nematodes studied herein (1–2 copies, Fig 3b and Figure S7 in S1 Supporting Information). Phylogenetic analysis revealed that paralogous EC-SOD genes in *A*. *cantonensis* might likely arise via two evolutionary events (Fig 3b), reflected by three paralogs (cluster I) in one clade with other nematodes and eight paralogs in another clade (cluster II, Fig 3b and Figure S7 in S1 Supporting Information). In addition, transcription analysis revealed two expression patterns occurred in these two clusters (Fig 3b). The genes in cluster I were relatively highly upregulated in the mammalian stage of *A*. *cantonensis* (L4 and L5 in Fig 3b), indicating these genes may be related to defend against definitive host-derived ROS. We examined the gene expression level of EC-SOD in *A*. *cantonensis* recovered from its nonpermissive host (mice) and permissive host (rat) using qPCR. We observed significantly higher transcription of the gene in cluster I (termed *P51547-D2* herein) in *A*. *cantonensis* collected from rats compared with the one harvested from mice using qPCR (Figure S11 in S1 Supporting Information).

In contrast, the EC-SODs in cluster II (six out of eight) were transcribed at significantly higher levels in L3, recovered from infected intermediate host compared with the other developmental stages (Fig 3b and Figure S10 in S1 Supporting Information), suggesting that these genes might be related specifically to parasite survival in the intermediate gastropod hosts. Excluding the genus *Angiostrongylus* from the phylum Nematoda, five well-studied and sequenced digenean trematodes (*S*. *japonicum*, *S*. *haematobium*, *S*. *mansoni*, *O*. *viverrini*, and *C*. *sinensis*), are gastropod-borne helminths that require snails as the intermediate host or first intermediate host [63]. We then analyzed and compared SODs of these five digenean trematodes and S. *mediterranea* (a free-living planarian) to determine whether some similarities existed among EC-SODs from the gastropod-borne helminths. Interestingly, the phylogenetic analyses showed that while EC-SOD in cluster II and the EC-SODs from parasitic flukes clustered into one clade, in which only contained the gastropod-borne flatworms and nematode (*A*. *cantonensis*) (GBH, Fig 3b and Figure S7 in S1 Supporting Information), which conflicts with the species topology (Fig 3a). Further, both the maximum-likelihood and Bayesian analyses recovered trees in which EC-SOD in cluster II and EC-SODs from digenean trematodes grouped together in one clade based on the conserved domain (“Sod_Cu”, Figure S8 in S1 Supporting Information). The multiple sequence alignment of the EC-SODs from 14 species showed some over-represented amino acid sites existed in the most members in the GBH clade (Fig 3c). Further, we extended the examination in 62 species (43 nematodes including 2 gastropod-borne nematodes, and 19 flatworms including 12 gastropod-borne flukes) and detected five amino acid sites that were significantly enriched in EC-SODs from the gastropod-borne helminths (Fig 3d, adjust *P*-value < 1e-5). This finding suggests that EC-SOD from cluster II may experience a convergent evolution at some sites with the EC-SODs of flukes.

### Proteases related to hemoglobin and tissue digestion in *A*. *cantonensis*

**Fig 4:**
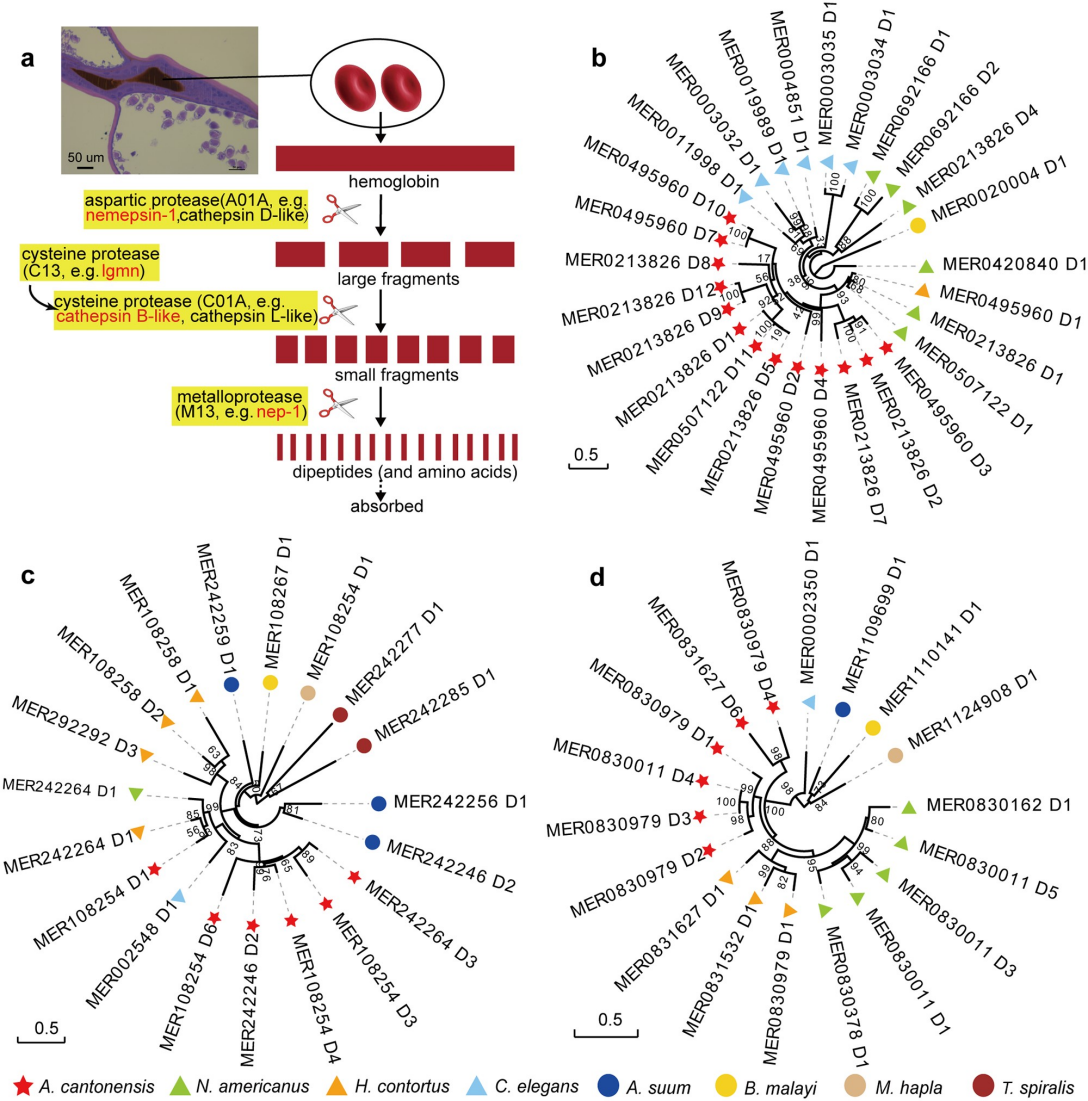
Phylogenomic analysis of three proteases related to hemoglobin digestion in eight nematodes. **(a),** H&E stained longitudinal section of the digestive tract from a female adult (on the upper left) shows the presence of red blood cells. The possible hemoglobin digestion pathway in the nematodes is illustrated on the right panel [65] with subfamily of enzymes (aspartic protease, cysteine protease and metalloprotease) related to hemoglobin and/or tissue digestion highlighted in yellow background. Specifically, the expanded subfamilies of enzymes from *A*. *cantonensis* are highlighted in red. Maximum likelihood phylogenies of *necepsin-1* (b), *Lgmn* (c) and *Nep-1*(d) show expansion of these proteases in *A*. *cantonensis*, *N*. *americanus* and/or *H*. *contortus*, all of which are blood-feeding nematodes.

Adults of *A*. *cantonensis* dwell in the pulmonary arteries of the definitive host, where worms mature and lay eggs. The parasite digests blood and other tissue components of the host for major protein synthesis [64] (Fig 4a). Hematophagous nematodes employ an ordered pathway with distinct proteases to degrade host hemoglobin or other serum proteins [65] (Fig 4a). Through annotation and comparative analysis of eight nematode genomes, we found that genes encoding proteases such as nematode-specific aspartic proteases (e.g., necepsin-1), cathepsin B-like, legumain (*Lgmn*) and neprilysin (e.g., NEP-1), inferred to be involved in hemoglobin digestion, are expanded in *A*. *cantonensis* (Fig 4 and Figures S13–16 in S1 Supporting Information). For instance, necepsin-1 (known as APR-2 in *N*. *americanus*) belongs to the aspartic proteases (MEROPS: A01A), and it is likely involved in the initial cleavage of hemoglobin [66]. Cysteine peptidases (including cathepsin B-like proteases) are likely involved in the second step of digestion. In addition, *A*. *cantonensis* has at least six genes encoding legumain, which likely activate cysteine proteases by specific hydrolysis of peptide bonds following asparagine residues [67]. In the final digestion step, metalloproteases, such as NEP-1, likely degrade small peptide fragments to dipeptides. Interestingly, the proteases necepsin-1, cathepsin B-like, legumain and NEP-1 are also expanded in *N*. *americanus* and/or *H*. *contortus* with respect to the five other nematodes studied herein (Fig 4, Figures S13–16 in S1 Supporting Information).

### Expansion of astacin-like genes in *A*. *cantonensis*

**Fig 5:**
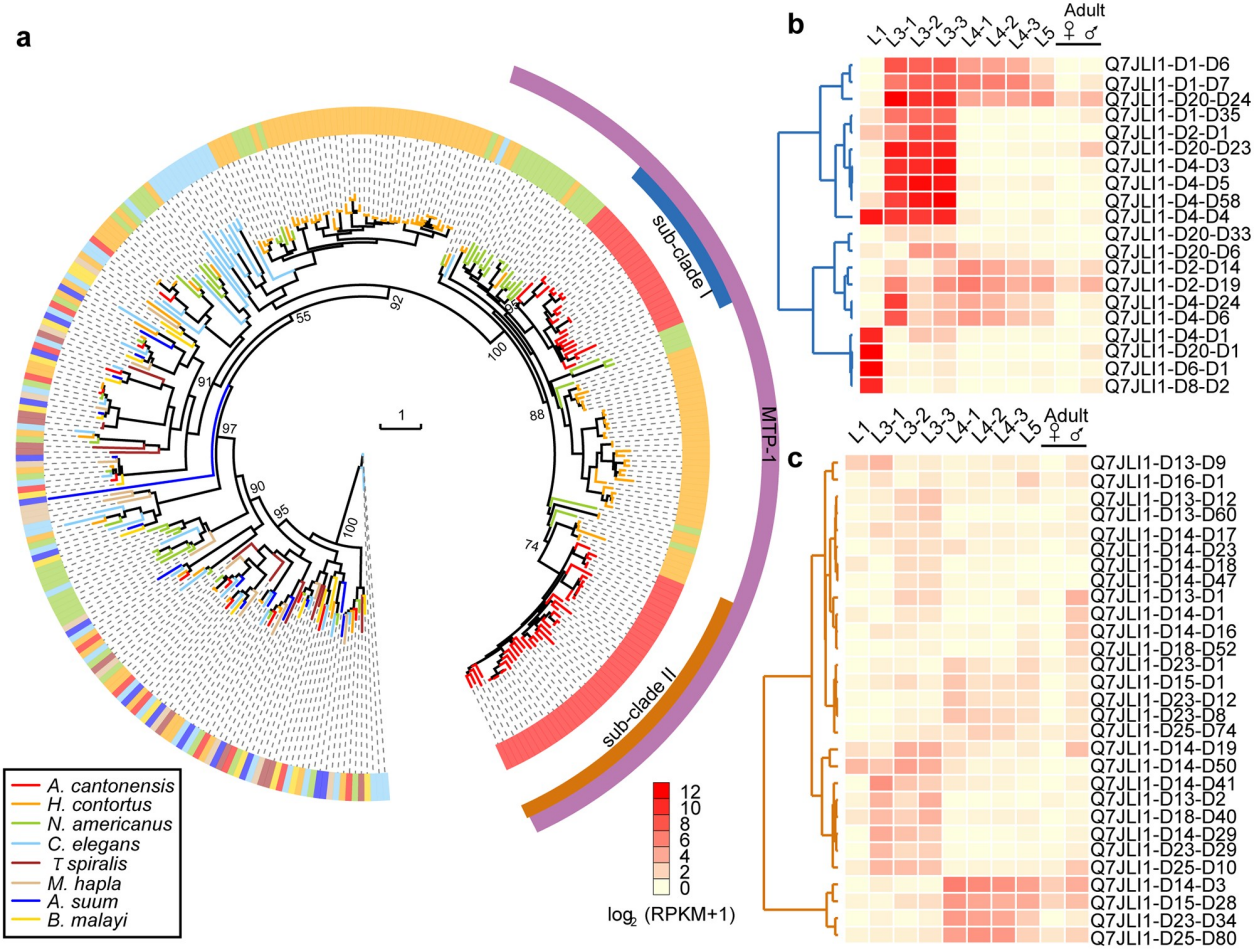
Evolution of astacin-like genes and expression pattern across in the life-cycles of *A*. *cantonensis*. **(a),** Phylogenetic analysis of the astacin-like genes containing the astacin domain (PF01400). We named the purple cluster MTP-1 because it shows the best hit with MTP-1 in the MEROPS database. **(b),** Expression patterns of expanded sub-clade I astacin-like genes in *A*. *cantonensis*. The genes in sub-clade I are upregulated in L1 or L3, which are two stages of invasion into intermediate host or mammalian hosts. **c,** mRNA expression pattern of expanded sub-clade II astacin-like genes in *A*. *cantonensis*.

Astacin-like metalloproteases are involved in molting, feeding and/or host tissue penetration in nematodes [68–70]. The number of genes encoding astacin-like protease (M12A, metalloproteases) in *A*. *cantonensis* (n = 75) was greater than that in *C*. *elegans* (n = 40). One subfamily, with the highest sequence similarity to the MTP-1 of *Ancylostoma caninum* [71], had only one gene in *C*. *elegans*, but had 63 genes in *A*. *cantonensis*. The expanded MTP-1 genes of *A*. *cantonensis* separated into two large subclades (subclade I: n = 22; subclade II: n = 41; Fig 5a) and ten amino acid sites that were relatively specifically between the two subclades (Figure S19 in S1 Supporting Information). Additionally, genes in subclade I were mainly expressed in the L1 or L3, which was also supported by qPCR (Fig 5b and Figure S18 in S1 Supporting Information). The genes in subclade II had relatively low transcription compared with subclade I (Fig 5 and Figure S18 in S1 Supporting Information). MTP-1 has been reported to be associated with tissue migration in *A*. *caninum* [71], suggesting that the expanded MTP-1s may be related to the survival and/or infectivity of *A*. *cantonensis*. Additionally, the sequence divergence and distinct RNA expression of these two subclades suggest that *A*. *cantonensis* may have acquired multiple related abilities. A study has shown that an expansion of astacin-like genes was also discovered in *Strongyloides* and *Parastrongyloides* species [72] (Clade IV). But our phylogenetic analysis showed that the expanded astacin-like genes in *S*. *ratti* formed a single and distinct clade that diverged from the above mentioned MTP-1 clade (Figure S20 in S1 Supporting Information). Recently, another study of comparative analysis of the major parasitic worms also identified the expansion of astacin-like genes in the clades IVa, Vc and Vb [13] (including *A*. *cantonensis*), which is consistent with our findings.

## Discussion

Parasite adaptation to the host is a key factor for its success [73]. The increasing genomic data for parasitic worms provides resources to explore biological and genetic differences between free-living and parasitic nematodes of plants and animals, shedding light on genomic adaptions [74]. These resources also offer unique opportunities to explore the fundamental biology of parasitic helminths and to identify potential interventions for diseases caused by worms.

Here we sequenced and assembled a high-quality reference genome of the Guangzhou strain of *A*. *cantonensis*, which were superior in quality to previous drafts for this species [13, 14] and published draft genomes for other strongylid nematodes [33–35]. We also employed transcriptomic data from multiple different developmental stages to reliably predict protein-coding genes and to underpin the subsequent analyses. We found that some of the genomic elements experienced multiple waves of expansion in *A*. *cantonensis*, including non-coding regions (e.g., RTE-RTE retrotransposons) and protein coding genes (e.g. EC-SOD and astacin-like genes). The paralogs of EC-SODs and astacin-like genes from different sub-clades, have different expression patterns in the molluscan stage and mammalian stage. Thus, the results may partly explain the adaptive evolution of the complex life cycle of *A*. *cantonensis*, such as the two different parasitic environments (mollusc and definitive rodent host).

Extracellular/secreted SOD of helminth parasite is one of the main components in excretory-secretory (ES) and plays a key role in fighting against host-produced ROS [60]. Our study showed that the cluster I EC-SODs of *A*. *cantonensis* mainly expressed in the mammalian stage, and expressed higher in the permissive host (rat) than in non-permissive host (mice). A previous investigation showed the high activity of EC-SOD in ES from rat-originated *A*. *cantonensis* [75]. And another study showed the higher SOD activity in *Heligmosomoides polygyrus* (mice is non-permissive hosts) than in *Nippostrongylus brasiliensis* (mice is non-permissive hosts) when they infected the mice [76]. These results suggested that some of parasitic nematode EC-SOD may be important for its survival in permissive mammalian hosts.

In contrast, the cluster II EC-SODs of *A*. *cantonensis* showed significant higher expression in the L3 (mollusc-dwelling). Moreover, the cluster II EC-SODs may experience convergent evolution at several amino acids with the EC-SODs of flukes. In *Fasciola hepatica*, EC-SOD was also identified in ES products from intra-molluscan larval stages [77]. The SOD showed the most significant differential expression patterns of three antioxidant enzymes (SOD, glutathione peroxidase, glutathione-S-transferase) in *S*. *mansoni* recovered from the susceptible snail than that from resistant snail [78]. Taken together, sequence convergence and expression similarity suggested the EC-SODs from gastropod-borne helminths might be related to their survival in gastropod species. Further, we observed that the liver flukes (*O*. *viverrini*: the family Bithyniidae and *C*. *sinensis*: the family Thiaridae and Bithyniidae) [79] with 3–4 copies of EC-SODs have a relatively broad spectrum of intermediate snail hosts than blood flukes (*S*. *japonicum*: *Oncomelania hupensis*, *S*. *haematobium*: the genus *Bulinus* and *S*. *mansoni*: the genus *Biomphalaria*) [63] with 1–2 copies of EC-SODs. While *A*. *cantonensis* has 7 paralogs in the GBH clade and has the broadest spectrum of snail hosts (the order Gastropod) [12] among the six species in this study. The largest copy number and diverged EC-SODs in *A*. *cantonensis* may provide more resources as well as possibilities for it to escape from host immune attack, which may therefore be a potential explanation for its survival in a variety of intermediate hosts. We also discovered some proteases involved in Hb digestion that were expanded in *A*. *cantonensis* and the other two blood-feeding nematodes. This result reveals a comparable spectrum of essential proteases involved in hemoglobin and possible tissue digestion among three haematophagous nematodes (i.e. *A*. *cantonensis*, *N*. *americanus* and *H*. *contortus*). These two instances merit further investigations as they may provide clues to broad-spectrum intervention to not only *A*. *cantonensis* control but also other parasites control. The high-quality genome and abundant transcriptomes of *A*. *cantonensis* should provide a deeper exploration of the co-evolution in the complex life cycles and host adaptability for helminths, which can be used as a resource to identify regions of genetic diversity in this species and help to deeply understand the global-spread of angiostrongyliasis in order to explore novel anthelmintic agents and/or vaccines.

## Supporting information

S1 Supporting Information File containing all supporting figures and detailed methods. (DOC)

S2 Supporting Information File containing all supporting tables. (XLSX)
