# Supplementary material for: The genetic basis of adaptive evolution in parasitic environment from the Angiostrongylus cantonensis genome
Source: PLoS Negl Trop Dis. 2019 Nov 21;13(11):e0007846. doi: 10.1371/journal.pntd.0007846 (PMC6871775; doi:10.1371/journal.pntd.0007846)
Supplement: S1 Supporting Information — (DOC) [file pntd.0007846.s001.doc]

# Supplementary Information

# Supplementary Figures


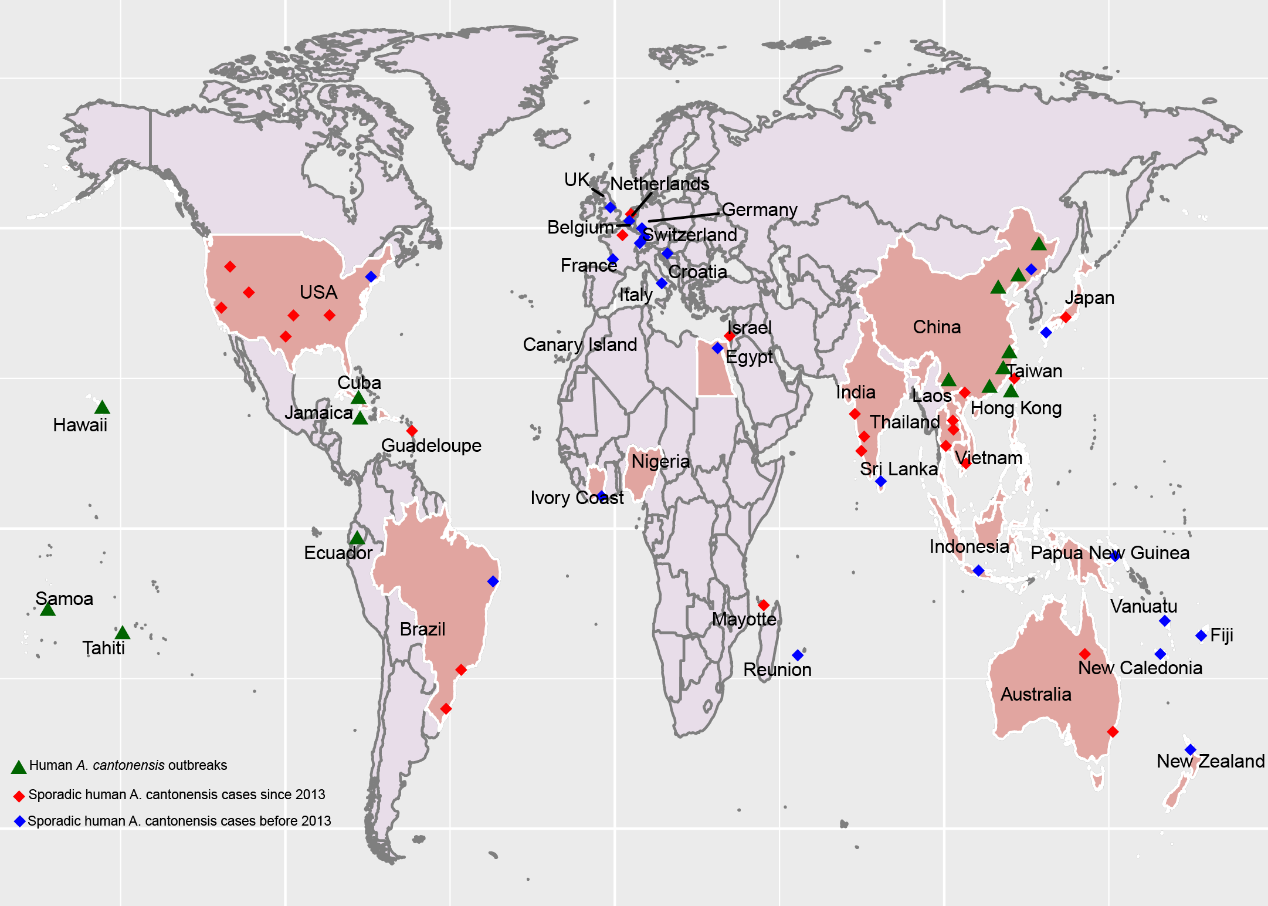


**Figure S1**. **The distribution of *A. cantonensis* and human *A. cantonensis* infections or outbreaks according to the literature**. According to the reviews [1-3] and recent case reports [4-11] (such as Paris, Texas, Israel, Guadeloupe), the marked countries (red) show the occurrence/prevalence (intermediate/definitive hosts infected with the larvae) countries of *A. cantonensis* according to the documents. Terrestrial and aquatic molluscs and definitive *Rattus* rat are involved in the spread of this pathogen. With the climate warming, global trade, and dietary habits (such as consumption of raw/uncooked snails), angiostrongyliasis is emerging worldwide [12]. The red rhombus shows cities with reported human cases since 2013. The blue rhombus shows cities with some reported cases before 2013. The green triangle shows reported outbreaks.


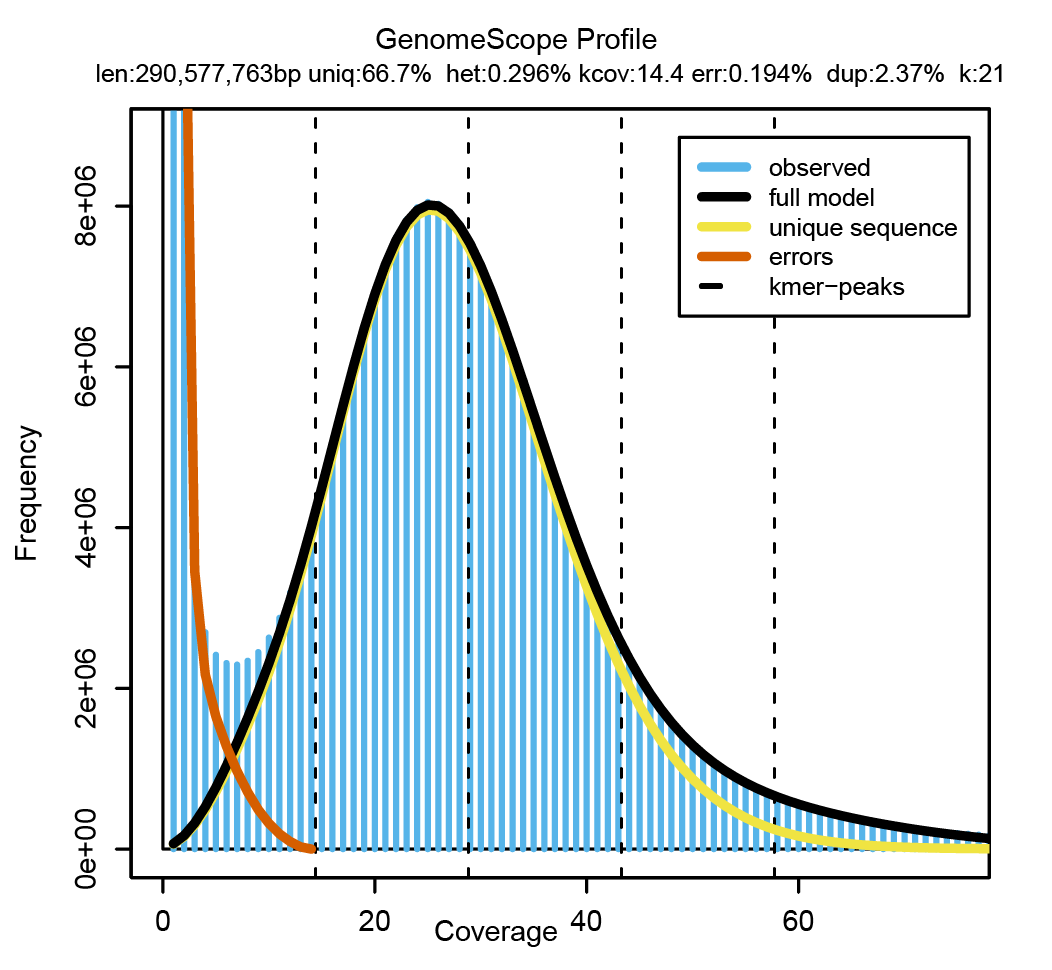


**Figure S2. Sequencing depth estimation of the *A. cantonensis* genome based on the Kmer-21 spectrum**. The genome size, heterozygosity and repeat content were estimated by GenomeScope [13] from Illumina sequencing reads (500 bp insert size).


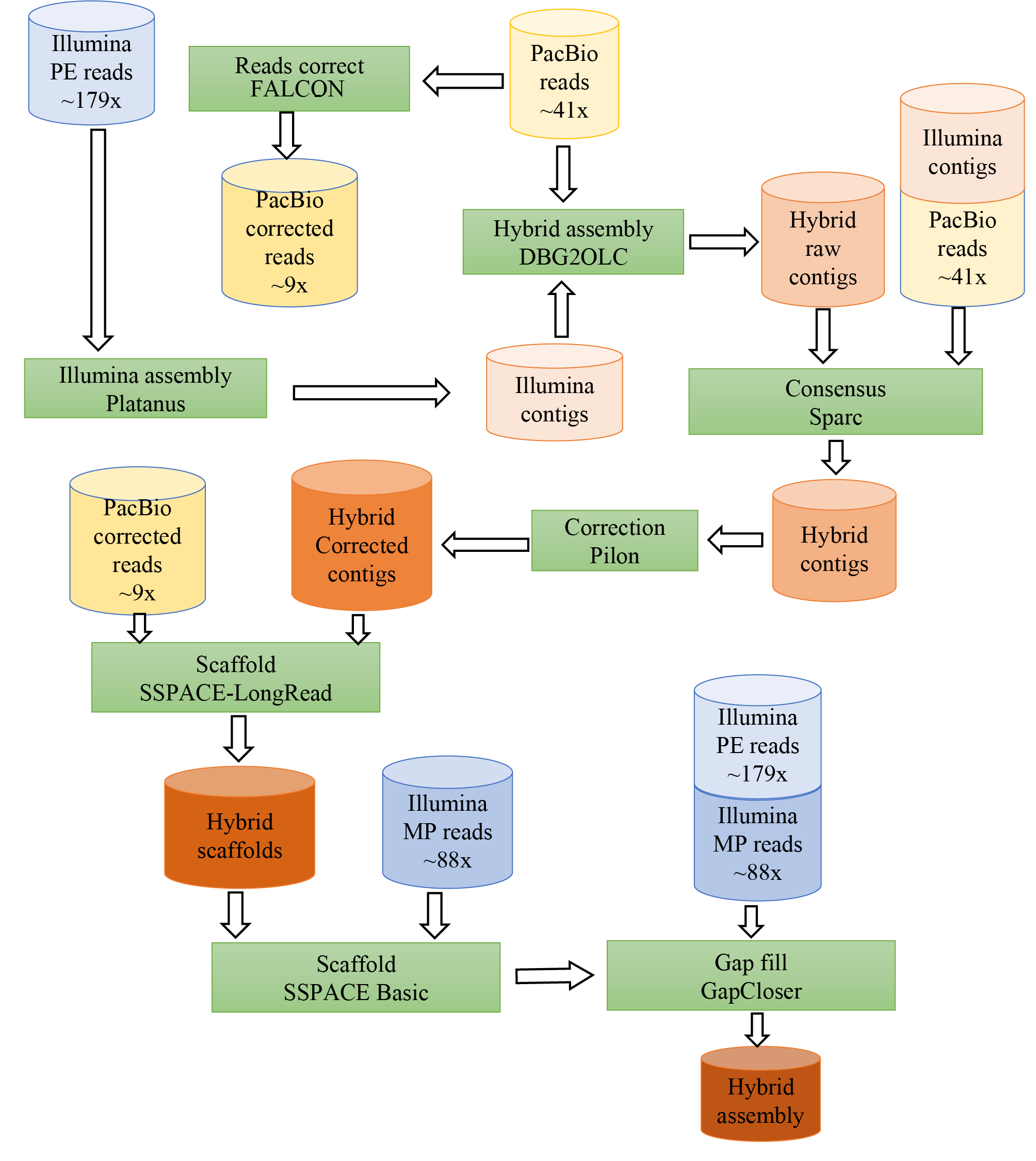


**Figure S3**. **The pipeline of hybrid assembly for *A. cantonensis* using Illumina and PacBio reads**. Approximately 179x Illumina paired-end reads (250, 500 and 800 bp libraries) were assembled into contigs with Platanus. The resulting Illumina contigs and PacBio raw reads were assembled with DBG2OLC into raw hybrid contigs. Then, Illumina contigs, PacBio reads and the raw hybrid contigs were used to call consensus with Sparc. Consensus sequences was corrected with Pilon using Illumina reads (paired-end and mate-paired). Pacbio reads corrected by FALCON and hybrid-corrected contigs were first scaffolded with SSPACE-LongRead. A second scaffolding was performed using Illumina mate-paired reads (2 kb, 5 kb, 10 kb and 20 kb) with SSPACE basic. Finally, GapCloser was used to fill gaps with Illumina reads.

**
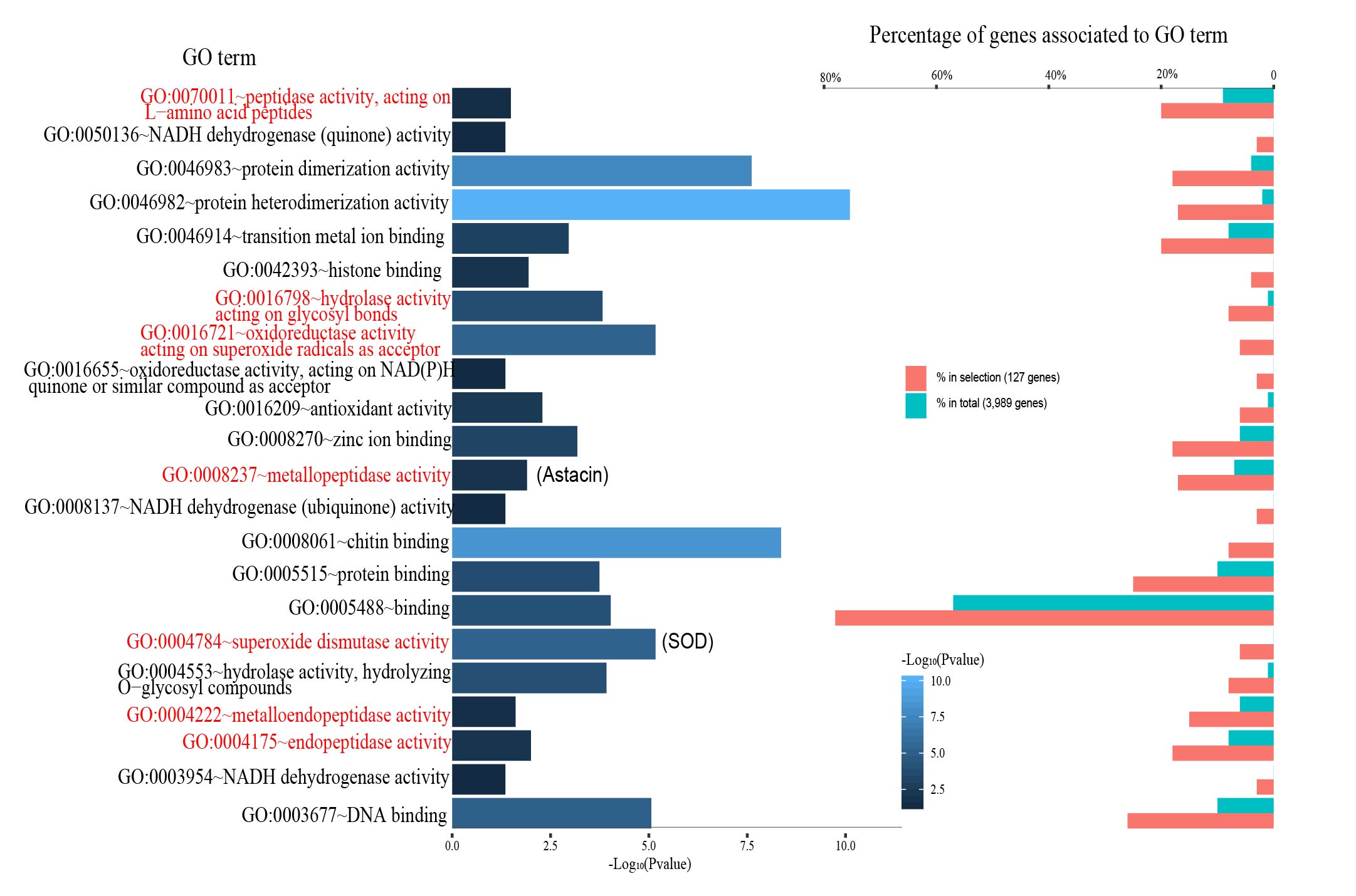
**

**Figure S4. GO enrichment of molecular function for ʺuniqueʺ gene families in *A. cantonensis* with corrected p-value ≤ 0.05.** Blue gradient colours indicated the corrected p-value for enrichment used with GO∷TermFinder. The ʺsuperoxide dismutase activityʺ and ʺmetallopeptidase activityʺ terms were significantly enriched.


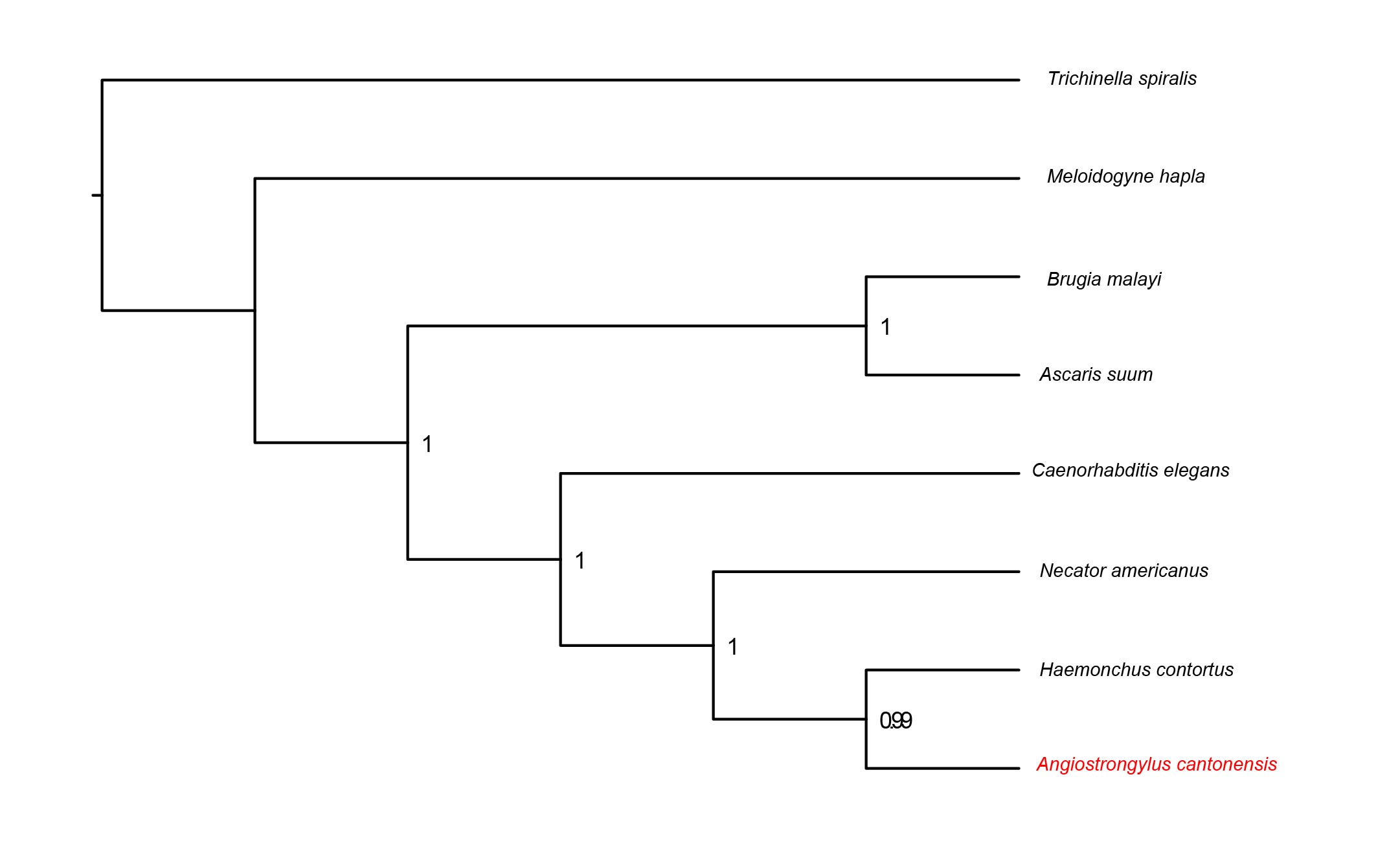


**Figure S5.** **Species tree based on** **ASTRAL method**. The ASTRAL tree is based on individual RAxML-estimated gene nucleotide trees (788). Tree is rooted with *T. spiralis*. Number in node are ASTRAL supporting value. The red labelled branch indicates the species study herein.


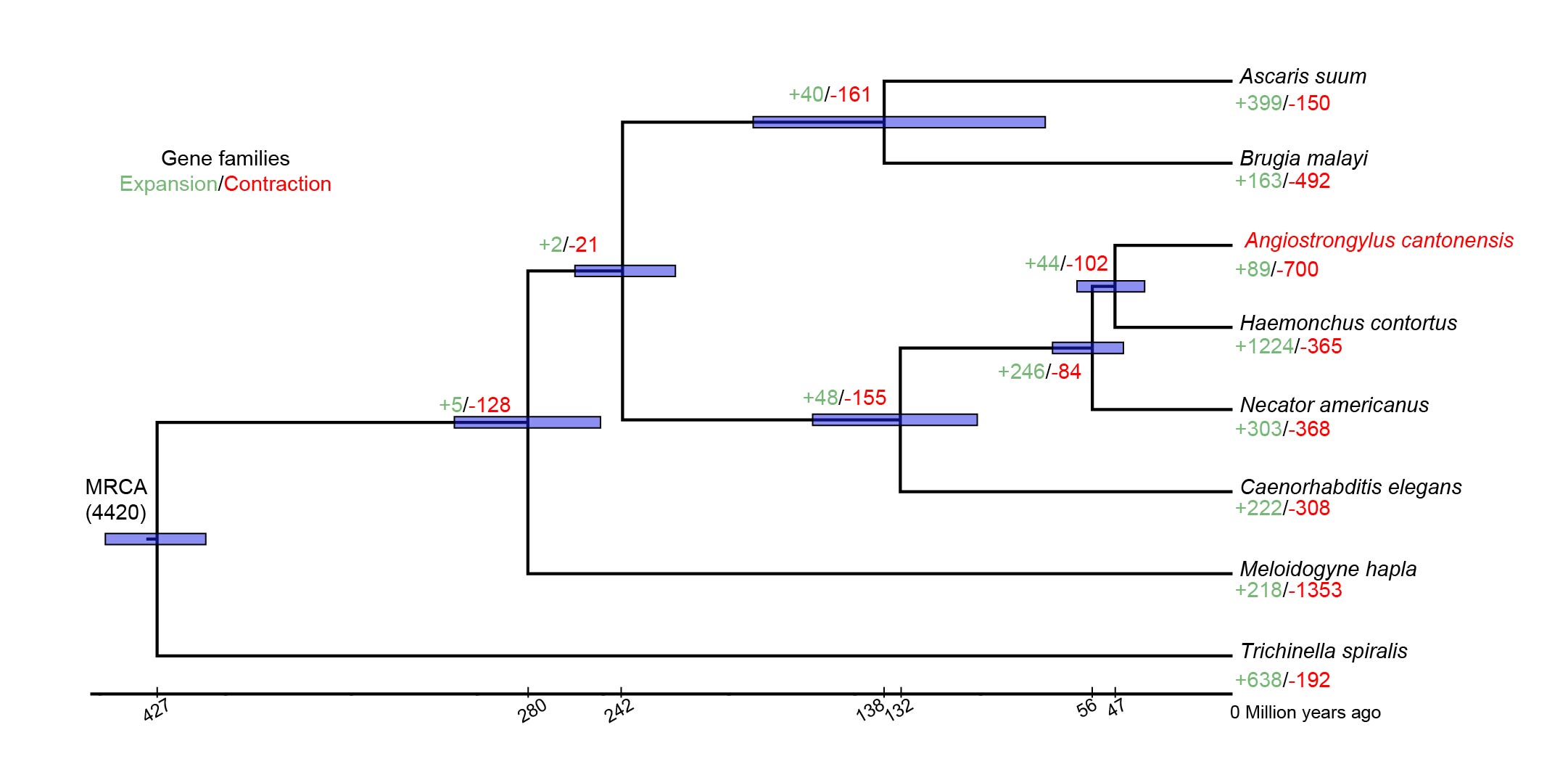


**Figure S6**. **The divergence time and expansion/contraction of gene families for eight nematodes**. Divergence times are estimated by MCMCTREE and indicated by light blue bars at the internodes; the range of these bars are the 95% confidence intervals of the divergence time. Numbers at the branches show the expansion and contraction of gene families. MRCA, most recent common ancestor. The clusters with single species were filtered in the gene family history analysis. The numbers in parentheses denote the number of gene families in the MRCA, as estimated by CAFE. The red labelled text indicates the species studied herein.


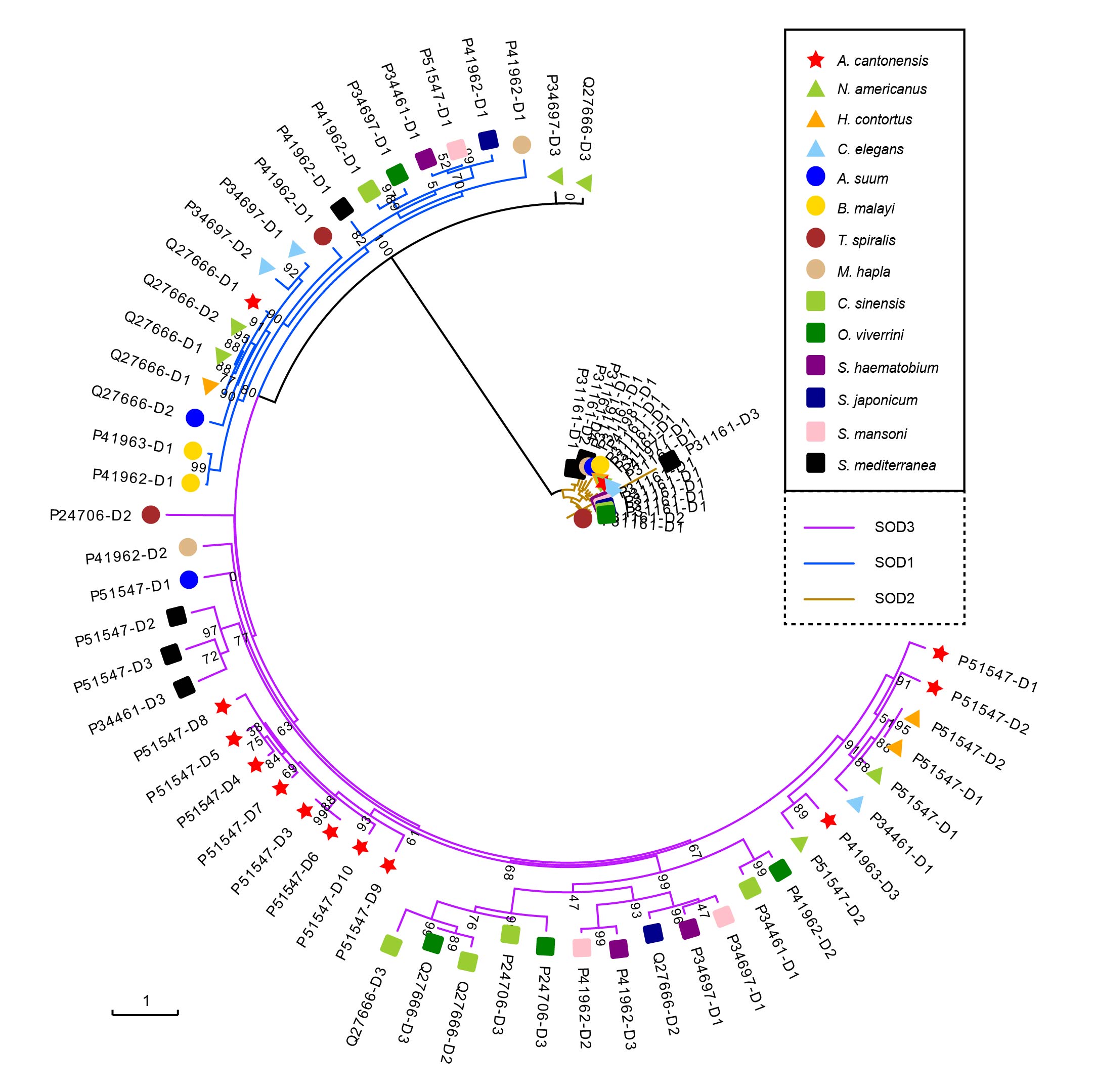


**Figure S7**. **The maximum likelihood phylogenetic tree of the SOD gene family in 14 species**. The SOD gene family contains three members (SOD1, SOD2 and SOD3). SOD3 is also named EC-SOD. Labelled text denotes the gene ID used in this study and the corresponding gene id used in their gene annotation file (Wormbase) is shown in S2 supporting information **Table S9**. The best model estimated by ProtTest was “WAG”.


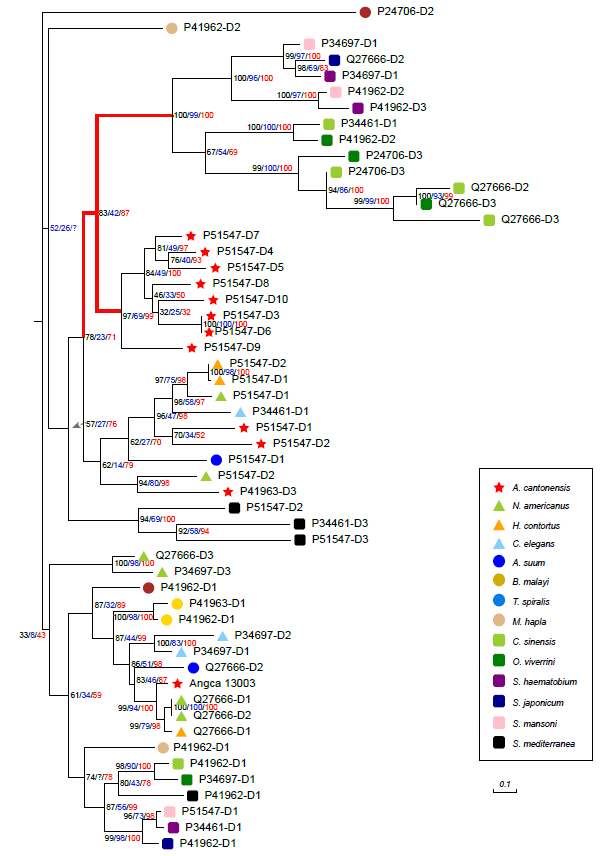


**Figure S8**. **Maximum-likelihood phylogeny of CuZnSOD based on the “Sod_Cu” domain (PF00080)**. Maximum likelihood and Bayesian methods were employed to infer the evolution of the CuZnSOD genes using IQTREE, RAxML and MrBayes, respectively. The Most topologies were the same using these three programs. Statistical support values in the nodes are bootstraps and percent of bayesian posterior probabilities (black - IQTREE; blue - RAxML; red - MrBayes). The amino acid substitution model was “WAG”.


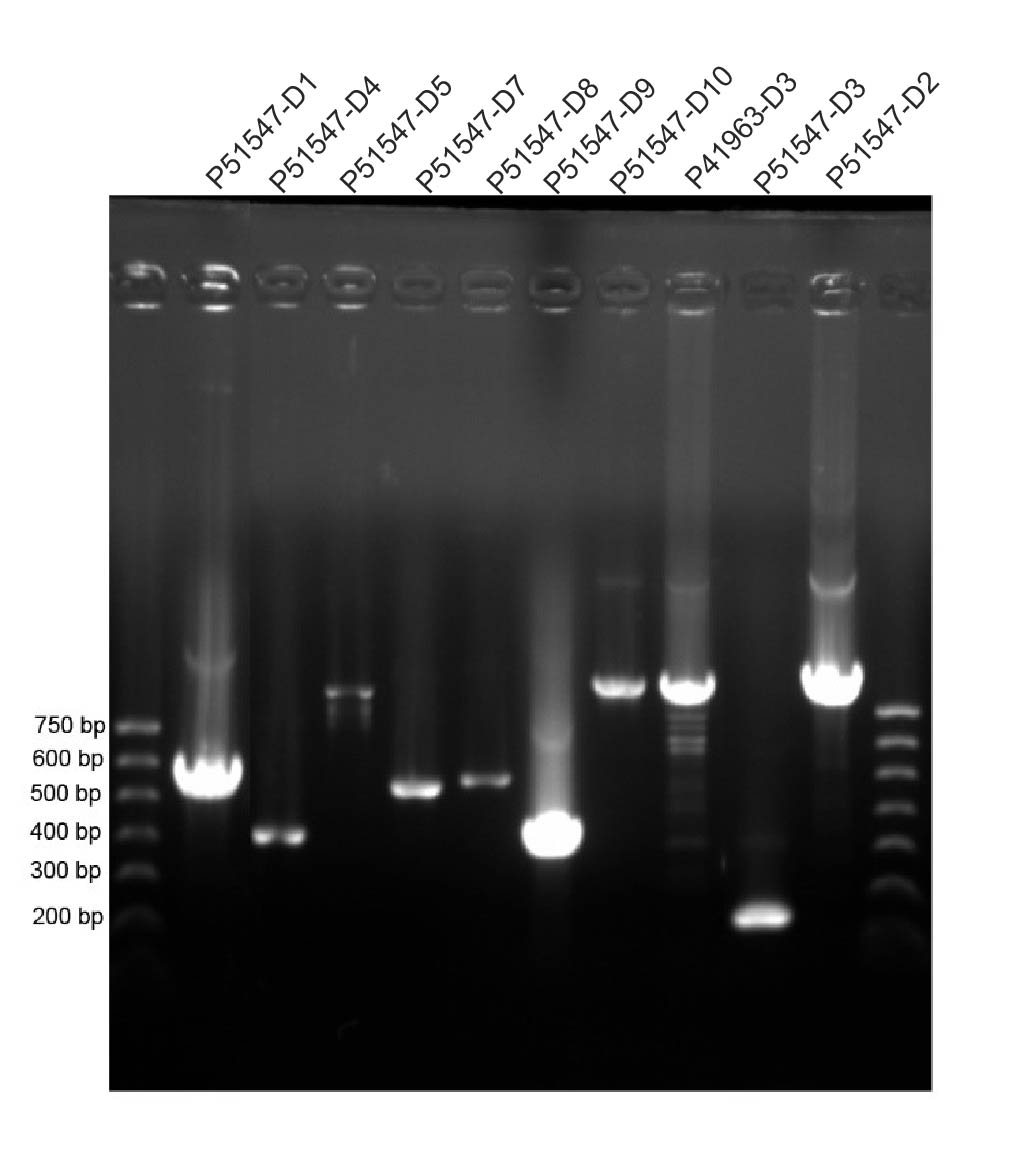


**Figure S9. PCR amplification of ten EC-SODs in the** ***A. cantonensis* genome.** The first and last lane are markers.


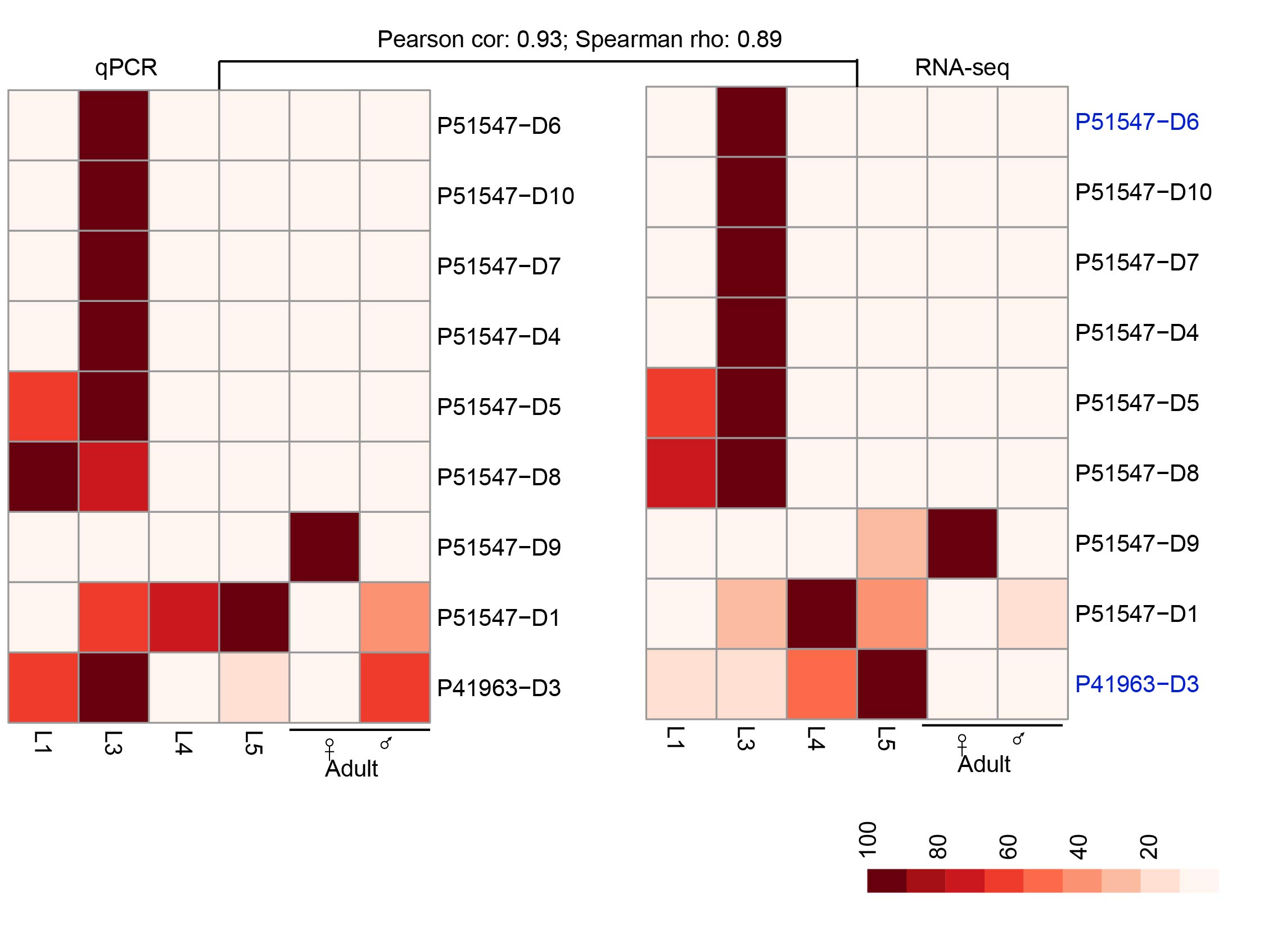


**Figure S10**. **Validation of RNA-seq results for expanded EC-SOD genes in *A. cantonensis* by quantitative real time polymerase chain reaction(qPCR/qRT-PCR)**. Expression levels of 9 genes in six samples used in this study were detected by qPCR. For each gene, the maximum expression in a certain sample was set to 100, and the relative expression levels in the other samples were calculated according to this maximum level. The blue labelled text shows the maximal RPKM was less than 10 across samples. The Pearson and Spearman correlation of genes with maximum expression >10 in six RNA-seq samples were 0.93 and 0.89 respectively. The Pearson and Spearman correlation of all genes listed in the heatmap were 0.82 and 0.85 respectively.


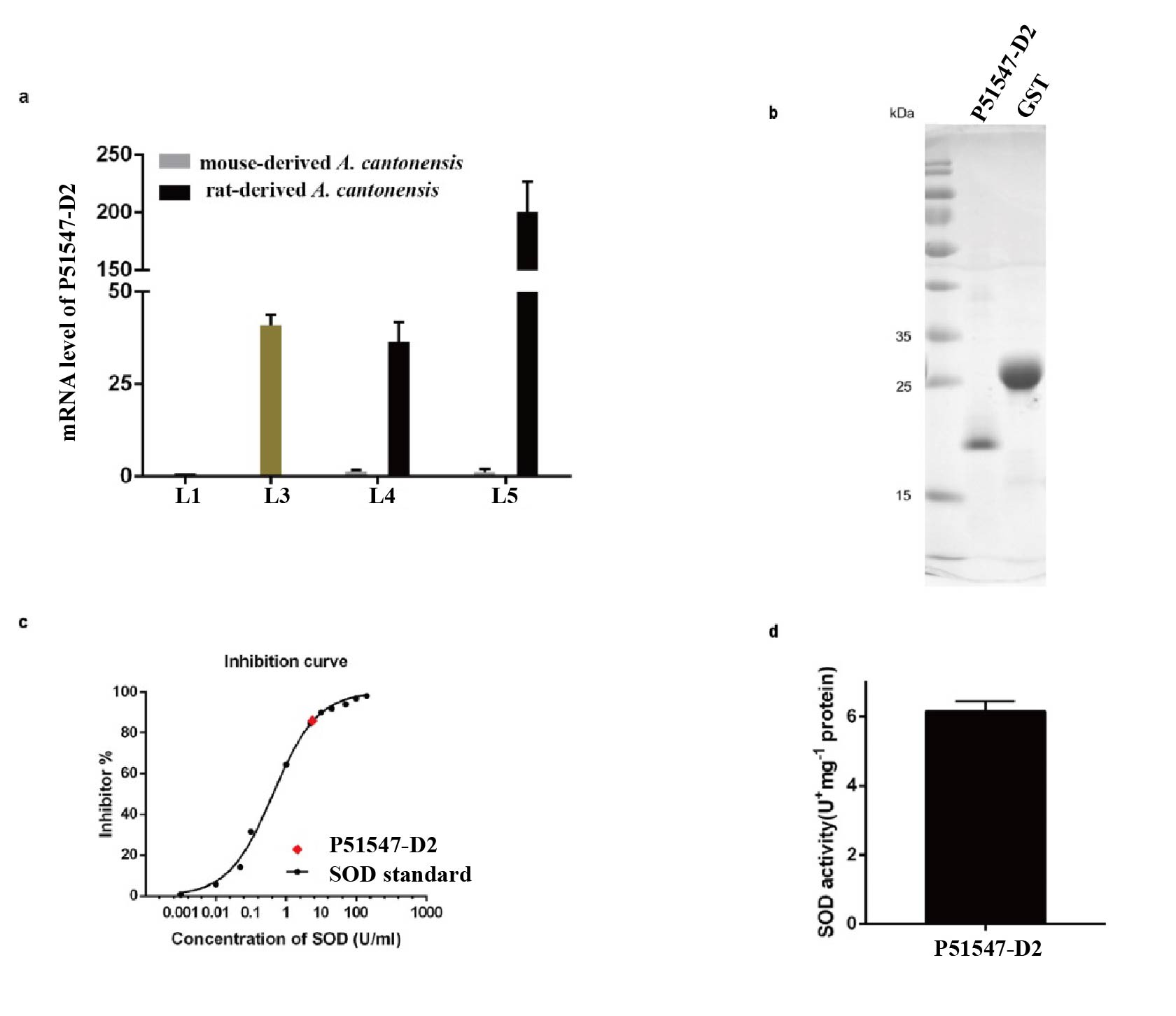


**Figure S11. Evidence for the potential functional SOD3 of *A. cantonensis* (P51547-D2) in terms of mRNA, protein and enzyme activity levels.** **a**, mRNA expression of P51547-D2 in developmental stages of *A. cantonensis* isolated from feces of rat, total tissue of infected snail, brain of rat, and mice, respectively, and lung vessel of rat (L1, L3, L4 and L5) by qPCR. We found a different expression pattern of P51547-D2 in *A. cantonensis* isolated from the infected definitive host (rat) and occasional host (mice). **b**, Purification of P51547-D2 protein and enzyme activity detection (**c**, **d**).


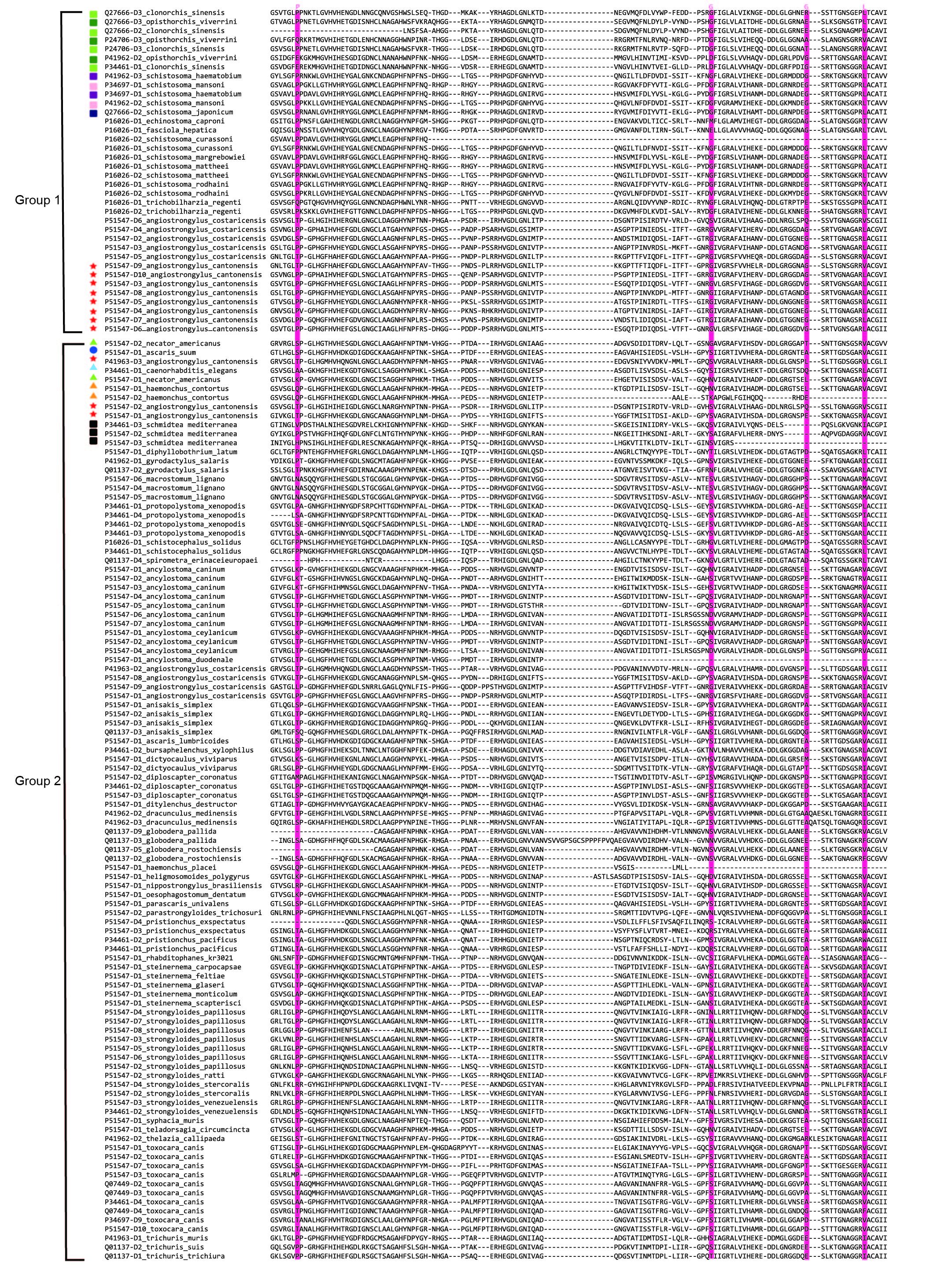


**Figure S12. Multiple sequence alignment of putative SOD3 in 62 helminths**. The red boxes depict over-represent amino acid positions in **Fig 3d** in the main text.


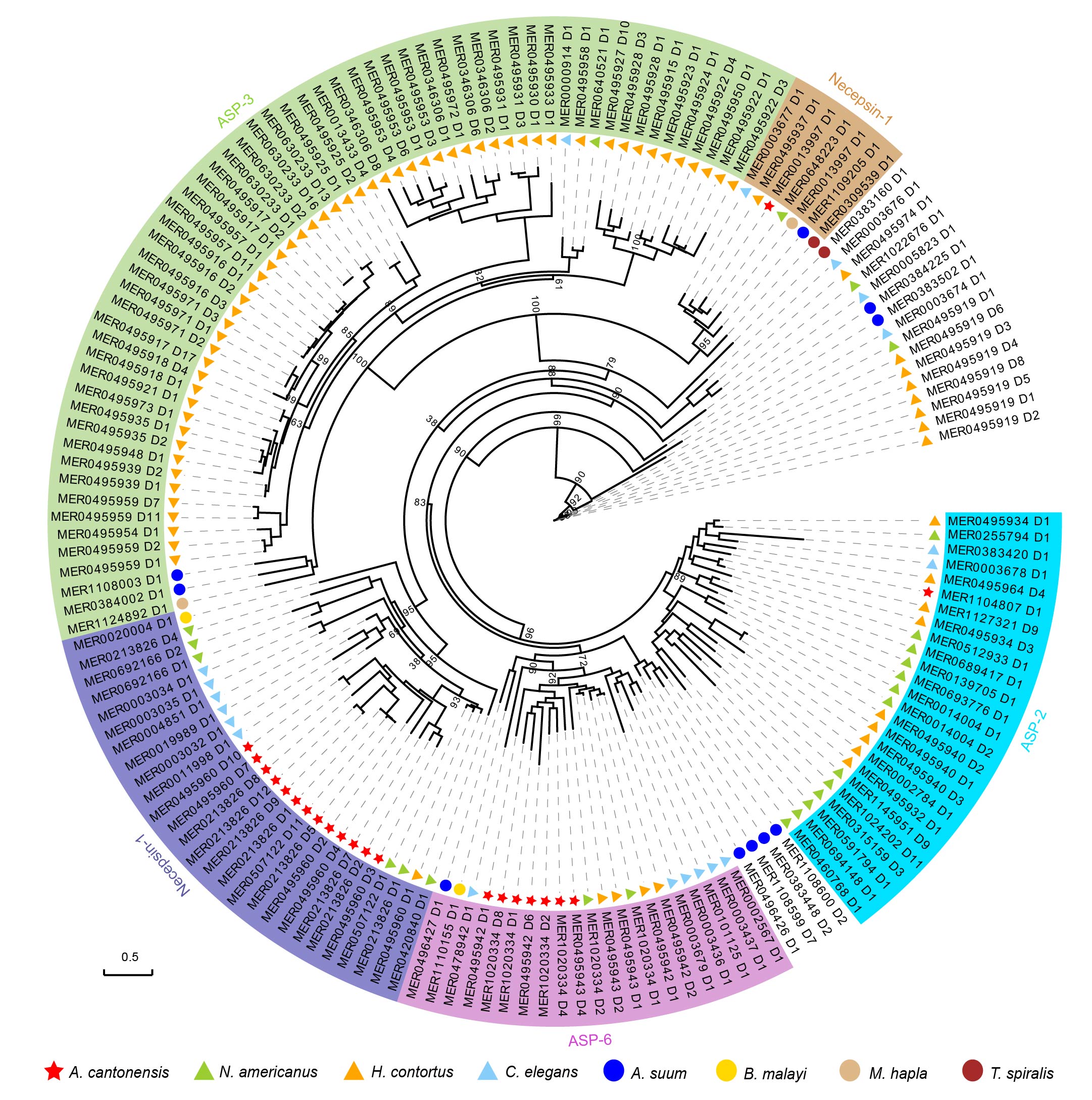


**Figure S13. Maximum-likelihood phylogenetic tree of A01 genes in *A. cantonensis* and other nematodes**. A01 is a family of aspartic peptidases in the MEROPS database. All genes contain a Pfam motif (PF00026, eukaryotic aspartyl protease). The colours of branch and leaf stand species. The best model estimated by ProtTest was ʺWAG+Gʺ.


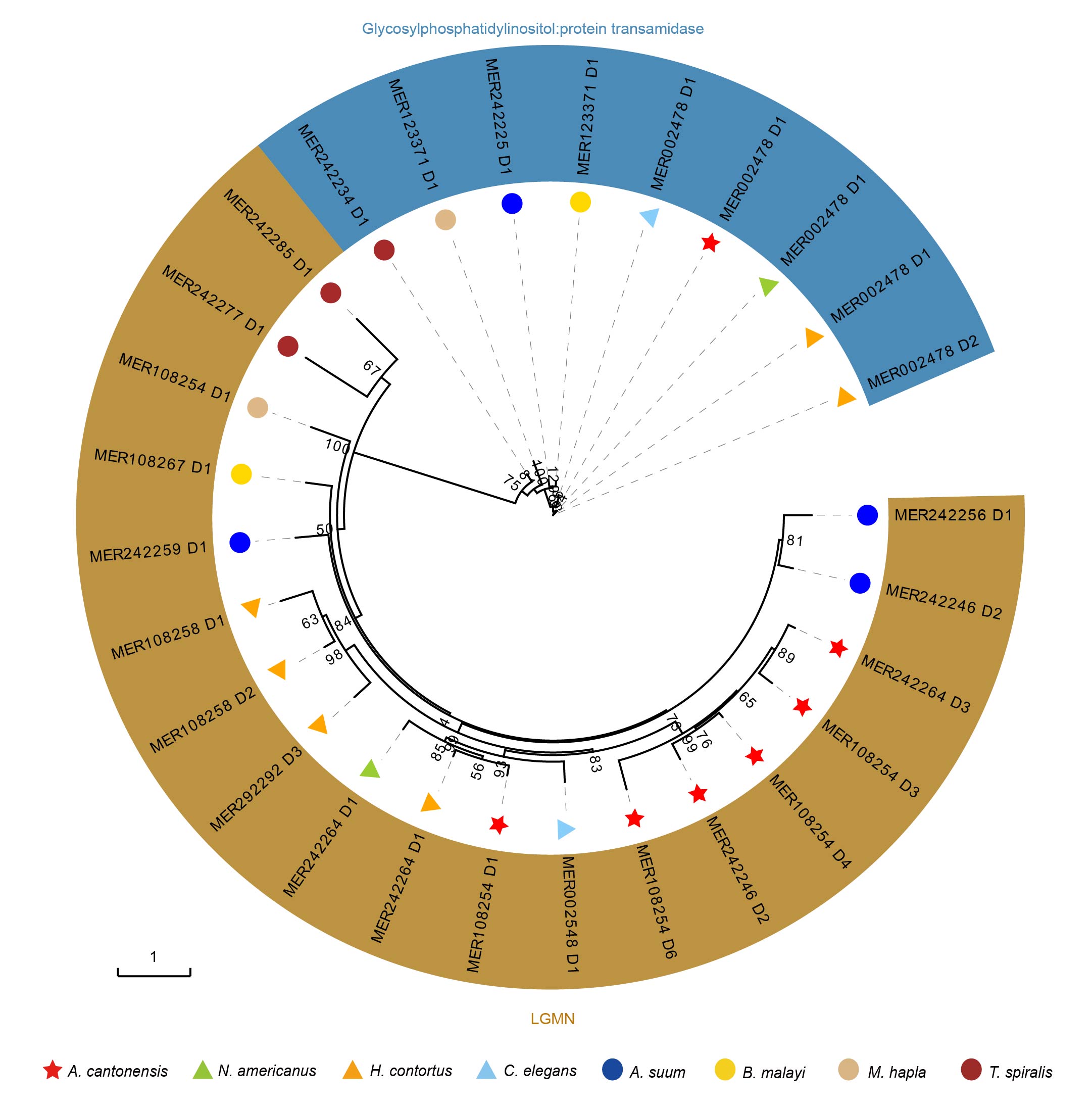


**Figure S14. Maximum-likelihood phylogenetic tree of C13 genes in *A. cantonensis* and other nematodes**. C13 is a family of cysteine peptidases in the MEROPS database. All genes contain a Pfam motif (PF01650, peptidase C13 family). The colours of branch and leaf stand species. The best model estimated by ProtTest was ʺLG+IGʺ.


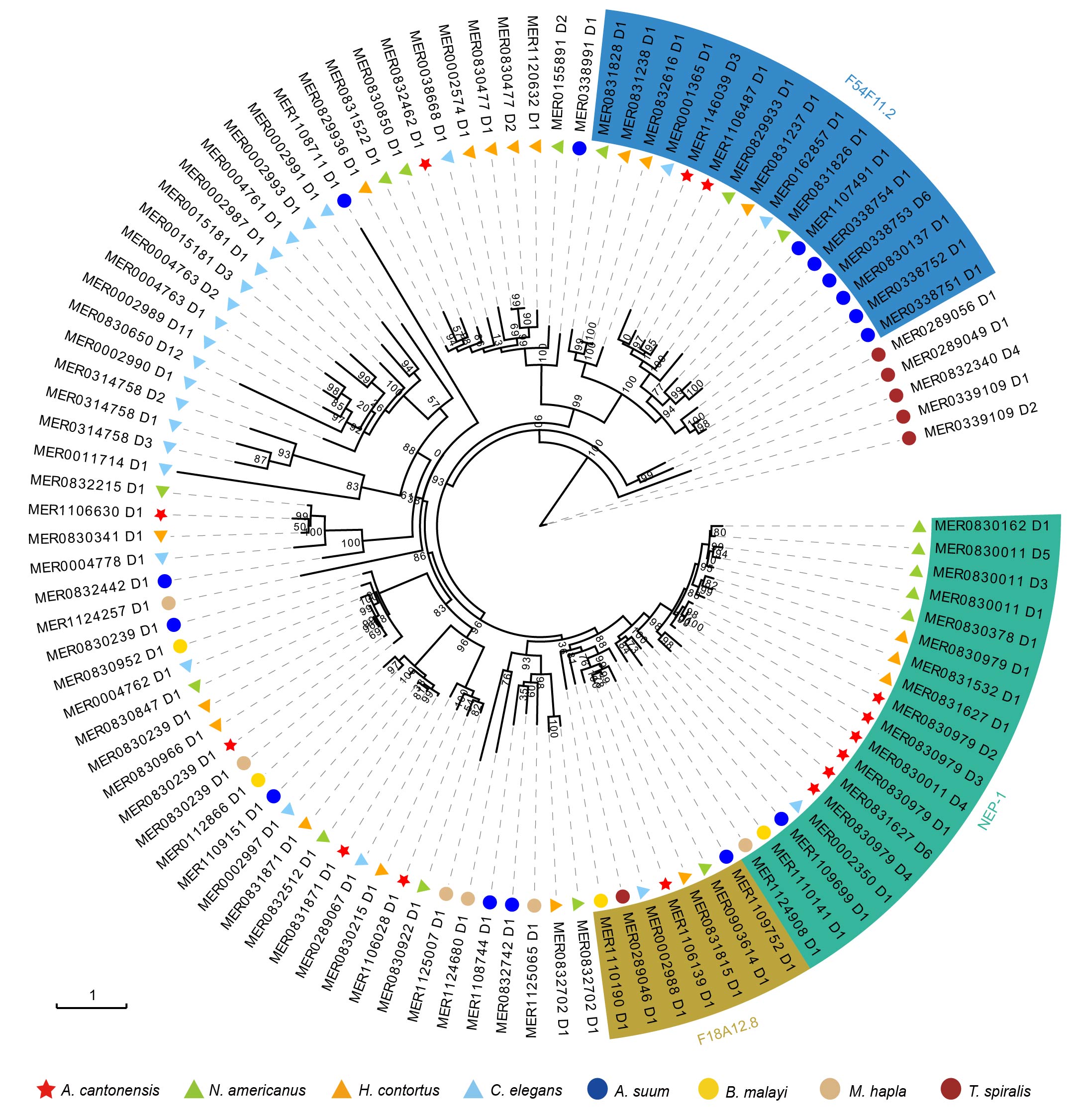


**Figure S15. Maximum-likelihood phylogenetic tree of M13 genes in *A. cantonensis* and other nematodes**. M13 is a family of metallo peptidases in the MEROPS database. The colours of branch and leaf stand species. The best model estimated by ProtTest was ʺLG+Gʺ.


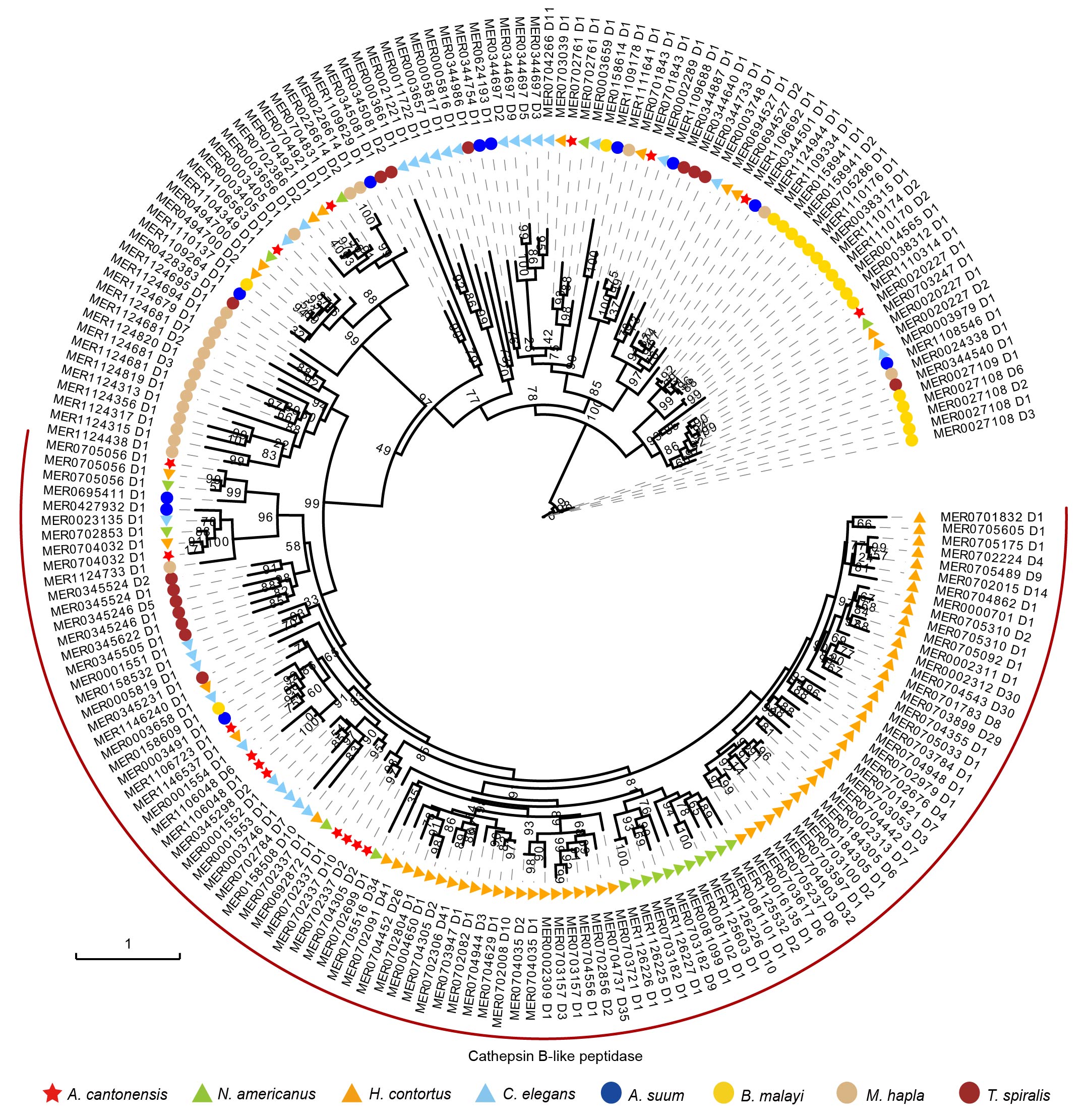


**Figure S16.** **Maximum-likelihood phylogenetic tree of C01 genes in *A. cantonensis* and other nematodes**. C01 is a family of cysteine protease in the MEROPS database. The colours of branch and leaf stand species. The best model estimated by ProtTest was ʺWAG+Gʺ.


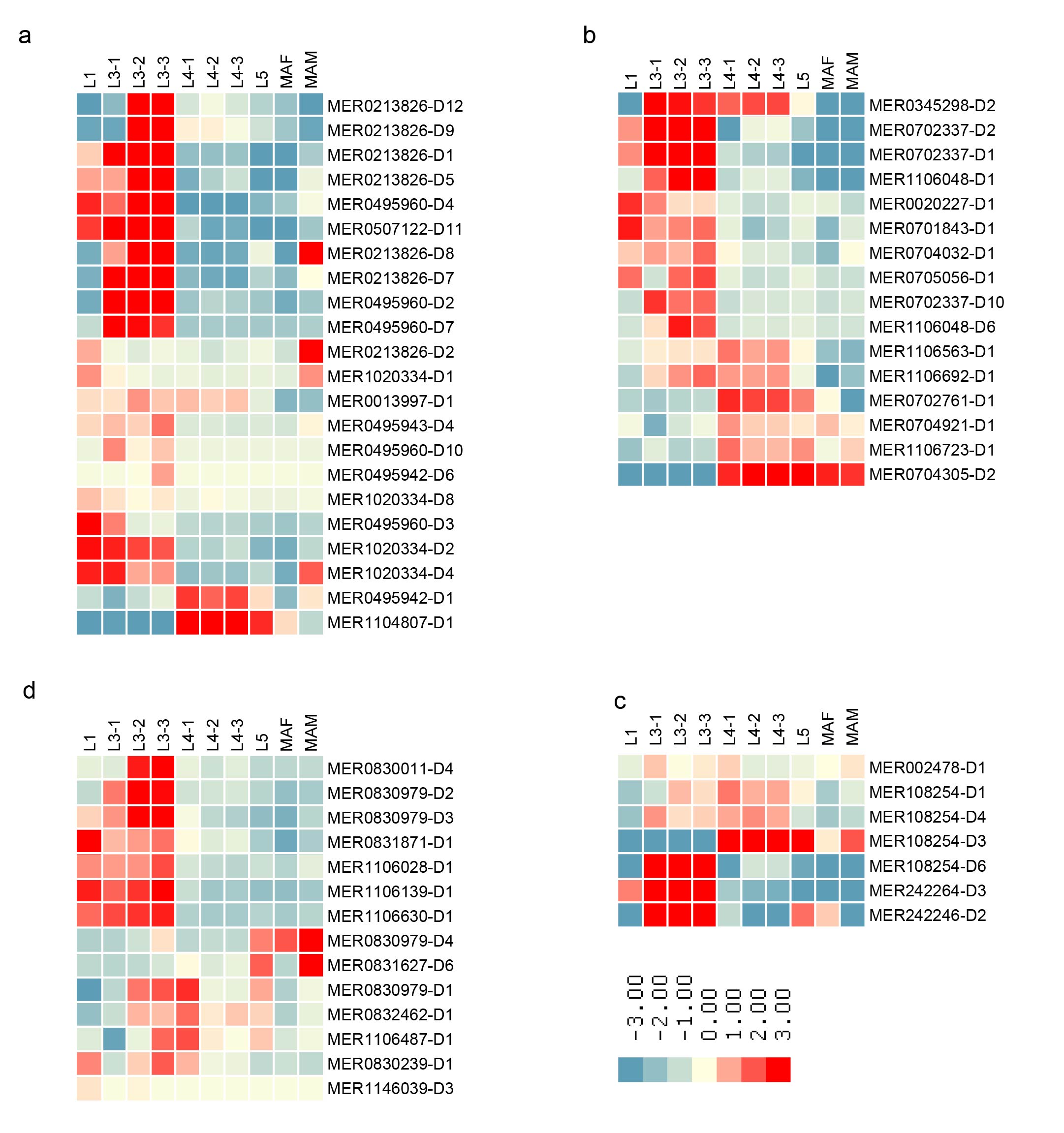


**Figure S17. The expression profiles of four proteases during the development of *A. cantonensis*.** a, b, c, and d are the expression pattern of A01, C01, M13 and C13, respectively. -1/-2/-3 indicate biological samples. Cluster3.0 was used to cluster. visualization performed with Treeview.


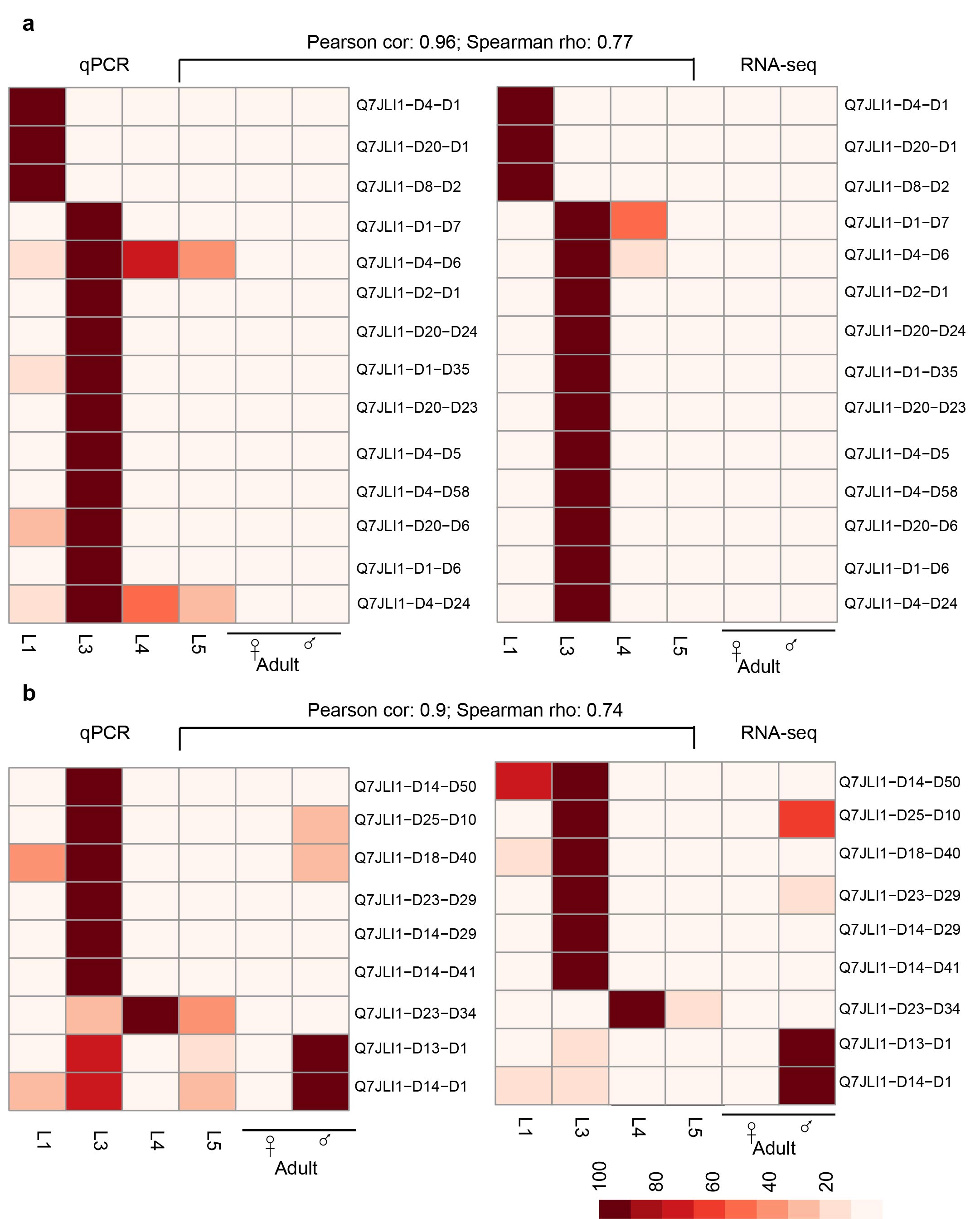


**Figure S18.** **Validation of RNA-seq results for expanded MTP-1 genes in *A. cantonensis* by qPCR**. The left shows expression detected by qPCR. The right shows RNA-seq expression. For each gene, the maximum expression in a certain sample was set to 100, and relative expression levels in the other samples were calculated according to this maximum level. (**a**) heatmap of genes from subclade I. The Pearson and Spearman correlation of genes were 0.96 and 0.77 respectively. (b) Heatmap of genes from subclade II. The Pearson and Spearman correlation of genes were 0.90 and 0.74 respectively. These results suggest a high reliability of highly expressed MTP-1 genes in our RNA-seq data.


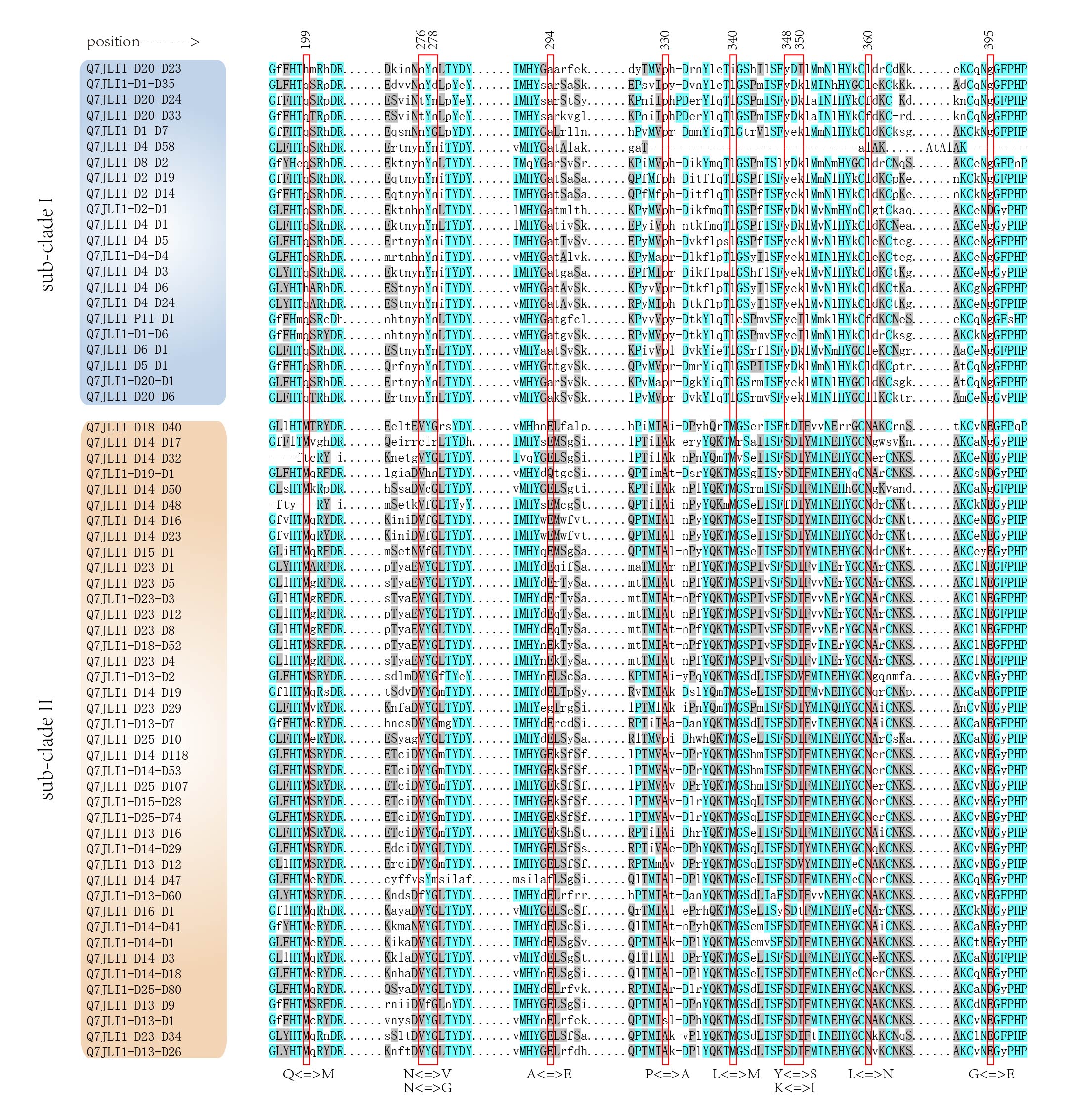


**Figure S19. Differential amino acids sites between two divergent astacin-like clades in *A. cantonensis* according to Fig 5a in the main text based on multiple sequence alignment**. Red boxes showed differential amino acids sites between sub-clade I and sub-clade II.


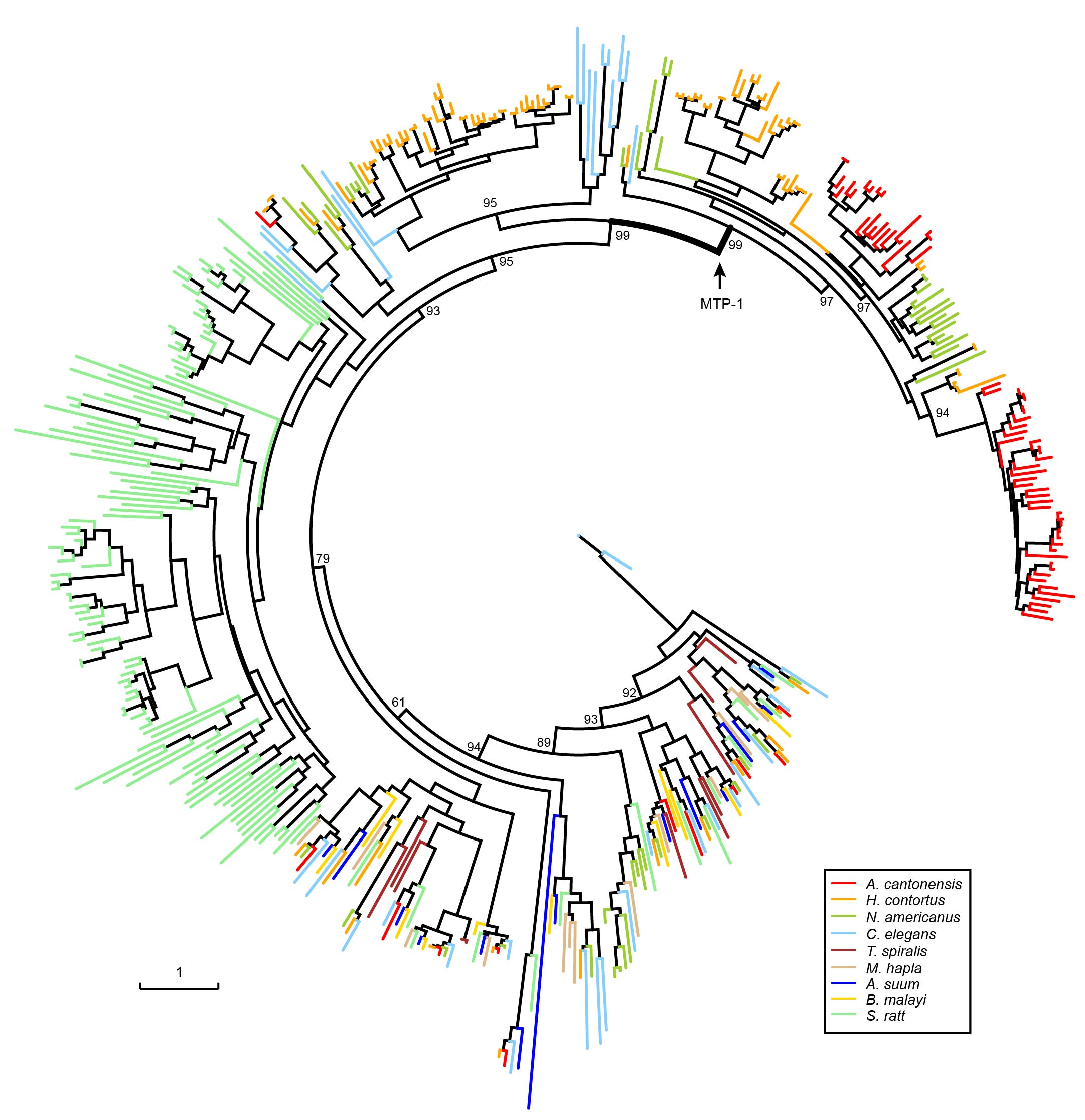


**Figure S20. The maximum likelihood phylogenetic tree of astacin-like genes in eight selected nematodes and *Strongyloides ratti***. The model “WAG+G” was used. The expanded astacins of *S. ratti* were distant from MTP-1. The members of MTP-1 of *A. cantonensis* fell into two branches. The colours of the branch depict different species.

# Supplementary Methods and Results

1. **Genome sequencing and assembly**

**1.1 Library preparation, sequencing and low-quality reads filtering**

Genomic DNA was extracted from ten female adults of *A. cantonensis* cultured in Guangzhou, China. Extracted DNA is fragmented by Covaris. For short-insert paired-end libraries (250 bp, 500 bp, and 800 bp), the fragmented DNA is combined with End Repair Mix, incubate at 20℃ for 30 min. Purify the end-repaired DNA with QIAquick PCR Puriification Kit (Qiagen), then add A-Tailing Mix, incubate at 37℃ for 30 min. Combine the purified Adenylate 3’ Ends DNA, Adapter and Ligation Mix , incubate the ligation reaction at 20℃ for 15 min. Adapter-ligated DNA is selected by running a 2% agarose gel to recover the target fragments. Purify the gel with QIAquick Gel Extraction kit (QIAGEN). Several rounds of PCR amplification with PCR Primer Cocktail and PCR Master Mix are performed to enrich the Adapter-ligated DNA framents. Then the PCR products are selected by running another 2% agarose gel to recover the target fragments.

For mate-pair libraries (2 kb, 5 kb, 10 kb and 20 kb), the fragmented DNA are end-paired with biotin dNTPs mix, and incubate at 20℃ for 30 min. Purify the end-repaired DNA with QIAquick PCR Purification Kit (Qiagen), target fragments are selected by running a 0.6% agarose gel. Then add Ligation Mix and incubate at 16℃ over night. The DNA fragments are circularized, and non-circularized DNA is removed by digestion. Circular DNA is fragmented (Covaris) and biotinylated fragments (corresponding to the ends of the original DNA ligated together) are purified by Dynabeads M-280 streptation (Invitrogen). Then add End-repaired Mix and , and incubate at 20℃ for 30 min. Purify the end-repaired DNA with streptavidin beads，then add A-Tailing Mix, incubate at 37℃ for 30 min. Combine the purified Adenylate 3'Ends DNA, Adapter and Ligation Mix, incubate the ligation reaction at 20℃ for 15 min. Purify the Adapter-ligated DNA. Several rounds of PCR amplification with PCR Primer Cocktail and PCR Master Mix are performed to enrich the Adapter-ligated DNA fragments. Then the 400-600bp PCR products are selected by running a 2% agarose gel.

The libraries were sequenced using the Illumina Hiseq 2000 platform. In total, we obtained ~100 Gb Illuminas raw reads (**Table S1**).

To obtain effective data for *de novo* assembly of the *A. cantonensis* genome, low-quality Illumina reads were filtered as the follows.

1. Reads with a percent of “N” more than 2% (paired-end libraries), 5% or 10% (mate-paired libraries)
2. Reads with a poly(A) structure.
3. Reads with low quality scores: Reads of paired-end libraries with 40% bases with quality scores ≤7; reads of mate-paired libraries with more than 30% or 40% bases with quality scores ≤7.
4. Reads with adapter contamination.
5. Filtered short insert-size libraries (250bp, 500 bp, 800 bp insertion size) with overlapping forward and reverse reads of >= 10 bp allowing 10% mismatches between the two ends.
6. PCR duplicates.

Finally, 77.56 Gb (approximately 267× coverage of the *A. cantonensis* genome) high-quality data were acquired.

In addition, a 20 kb library for PacBio sequencing was constructed using the SMRTBell Template Prep Kit 1.0 (Pacific Biosciences, http://www.pacb.com/), according to the manufacturer’s instructions. The SMRT Bell Template sequencing primer with DNA polymerase was applied to the SMRT Cell for the sequencing reaction. The P4 DNA polymerase with C2 chemistry (P4-C2, a total of 11 cells for the 20 kb library) was used in the sequencing reaction using the PacBio RS II sequencer (Pacific Biosciences). To obtain high-quality of subreads, we first filtered these reads with a size < 2 kb and RQ value < 0.8. A total of 11.95 Gb (~41-fold) of subread bases with a mean read length of 8.5 kb was generated from the 20 kb library. FALCON [14] (release July 2015) was used to correct these reads. Finally, a total of 2.7 Gb high-quality of error-corrected subreads bases was obtained.

- 1. **Genome size estimation**

The genome size of *A. cantonensis* was estimated based on the K-mer spectrum[15]. Given the K-mer frequency resembles the Poisson distribution, and the sufficient coverage, the genome size can be estimated by

Genome Size = K*_num_*/K*_depth_*

where K*_num_* is the number of K-mers and K*_depth_* is the expected depth of K-mers. In this study, we used JELLYFISH v2.1.1 [16] to count kmers (kmer=21), and GenomeScope [13] (<http://qb.cshl.edu/genomescope/>) (ʺ21 100 10,000ʺ) to estimate the genome size and heterozygosity (**Figure S2**). The estimated genome size was 290Mb and the heterozygosity was 2.9‰.

**1.3 Genome assembly and evaluations**

Considering medium coverage of the PacBio data, we employed a hybrid approach similarly to that described for the apple genome [17] (**Figure S3**): Illumina paired-end reads were firstly used to construct raw contigs with Platanus [18] (v1.2.4). PacBio subreads and Illumina contigs were imported into the DBG2OLC pipeline [19] (release in June 2015) to assemble backbone contigs with parameters ʺk 17 AdaptiveTh 0.001 KmerCovTh 2 MinOverlap 20 RemoveChimera 1ʺ. Then we used Sparc to call consensus. Due to the high error rate of long reads, we used Illumina paired-end reads for polishing with Pilon [20] (v1.22). Corrected PacBio long reads were used for scaffolding with SSPACE-LongRead[21] (v 1-1). Next, Illumina mate-paired reads with an insert size range from 2 kb to 20 kb were used for scaffolding with SSPACE Basic [22] (v2.0) in a step by step manner. Finally, Illumina reads were used to close gaps in the assembly with GapCloser implemented in SOAPdenovo2.

The quality of the assembly was evaluated by assessing assembled transcriptome data (**Table S2**), the CEGMA [23] (v2.4) and BUSCO [24] (v3.0) pipelines (**Table 1 in main text**).

For assembled transcripts, we used Trinity [25] (v2.4.0) for the *de novo* assembly of RNA-seqs data (L4, L5 and adult). A total of 33,945 transcripts longer than 200 bp were obtained and aligned to the assembly with BLAT aligner with default parameters. At least 95% of the transcripts could be identified in both assemblies with coverage ≥70%.

Finally, we found that at least 98% of the core eukaryotic genes (248 core eukaryotic genes), and 84% of the nematoda set (982 genes) were completely detected in the genome of *A. cantonensis* using the CEGMA and BUSCO pipelines, respectively (**Table 1 in the main text**).

Additionally, we compared the previously published assemblies with our assembly based on N50, gaps, and gene completeness using BUSCO and CEGMA (Table 1 in the main text). Our analyses suggested that the draft assembly of *A. cantonensis* herein was of high quality.

1. **Genome annotation**

**2.1 Gene model prediction and functional annotation**

Evidences from homolog-based and RNA-seq data were employed to generate gene models for *A. cantonensis*.

**Homolog-based prediction**: First, the protein sequences of *Caenorhabditis elegans*, *Ascaris suum*, *Haemonchus contortus*, and *Necator americanus* (Wormbase W246) were aligned to the *A. cantonensis* genome with TBLASTN to search for putative non-redundant protein sequences of each gene set with an e-value <1e-5 and conjoined high-scoring pairs (HSPs) for each gene pair by Solar (v0.9.6). Then Genewise [26] (v2.4.1) was used to define gene models.

**RNA-seq refinement**. The RNA-seq data provide a good supplement for gene prediction based on the homologous-based method, as most of open reading frames (ORFs) in the homologous-based gene models are not intact (lack start codon and/or stop codon). First, RNA-seq of different development stages (L1, L3, L4, L5 and adult) were mapped to the *A. cantonensis* genome with Tophat2 [27] (v2.08) with default parameters and assembled into transcripts using Cufflinks [28] (v2.2.1). The assembled transcripts were used to refine the gene models with the homolog-based approach that overlapping the gene models with RNA-seq and homology-based methods were merged.

A total of 13,473 gene models were obtained for the genome assembly of *A. cantonensis* using the above described methods.

We annotated the function of predicted gene models by aligning to the Swiss-Prot [29] (release Jun 2019), NCBI Nr (release Sep 2017), and KEGG [30] (release 89) databases. The gene symbols and pathways were assigned based on the best blast hit against the Swiss-Prot and KEGG databases. GO terms, motifs and domains of protein sequences were annotated using InterProScan [31] (release 5.3) by searching against publicly available databases, including Pfam, PRINTS, PANTHER, PROSITE, ProDom, and SMART.

**2.2 Transposable element analysis**

We predicted the repetitive elements using a combination of homology-based and *de novo* approaches.

**Homology-based repeats**. RepeatMasker (v3.3.0, <http://www.repeatmasker.org/>) [32] and RepeatProteinMask were employed to identify transposable elements (TEs) based on homologous search against a library of Repbase [33] (release 20.04) using the parameters ʺ-nolow -no_is -norna -parallel 1ʺ and ʺ-noLowSimple –pvalue 1e-4ʺ.

2) ***De novo* repeat library***. De novo* repeat prediction was conducted in two steps. First, the *ab initio* prediction programme Piler [34] (v1.0), Repeatscout [35] (v1.0.5) and LTR-FINDER (v1.0.6, <http://tlife.fudan.edu.cn/ltr_finder/> ) [36] were employed to build the *de novo* repeat library respectively. Second, putative protein-coding genes were removed from the library by alignment to the Swiss-Prot database. Third, de novo repeats from three predictions were merged. Finally, RepeatMasker was used to find repeat in the genome.

Approximately 54.61% (total 154.45 Mb) of the *A. cantonensis* genome is transposable elements (**Table S3**). Long interspersed elements (LINEs), covering approximately 39.17% (110.78 Mb) of the assembly, represented the most abundant class (**Table S4**). The most abundant TEs was RTE-RTE superfamily, belonging to LINE class, which accounted for 72% of the TEs.

**Transcriptome**

**3.1 RNA-seq analysis**

**RNA sequencing**. Seven libraries of four larval stages (L1, L3, L4 and L5) and the adult stage (female, male and mixed sex adults) were constructed using the Illumina TruSeq RNA sample preparation kit according to the manufacturer’s instructions. The libraries (insert size 200 bp, insert size of 100~500bp for L1 and L3 libraries) were sequenced in 90 bp using the paired-end model with the Illumina Hiseq 2000 platform. L3 and L4 are important stages for understanding mechanisms underlying the infectivity and pathogenicity of *A. cantonensis*. An additional four libraries (two biological replicates for L3 and L4,) were constructed using the TruSeq RNA Sample Prep Kit v2 according to the manufacturer’s instructions and sequenced using the Illumina Hiseq 4000 platform with a length of 100 bp and paired-end mode. We achieved 36~149 million reads per library.

**Gene expression quantification**. Reads were mapped to the genome with Tophat2 and subsequently analyzed with in-house Perl scripts. Of the reads, 75~87% could be uniquely mapped against the genome for each sample. We quantitated the gene expression level using unique mapping reads and normalization per kilobase of transcript per million mapped reads (RPKM) [37].

**Functional enrichment**. Gene Ontology enrichment analysis was conducted with the GO::TermFinder package. We performed KEGG pathway enrichment analysis with custom scripts using the hypergeometric test and the QVALUE in R was used to correct for multiple testing with a false discovery rate (FDR) cut-off of 0.01.

**3.2 Quantitative real-time polymerase chain reaction (qPCR) analysis of the transcription of EC-SOD and MTP-1 subclade I/II genes in *A. cantonensis* (total of 23 genes)**

L1s and L3s were collected from rat feces and digested tissue of *Biomphalaria glabrata* respectively. The larvae and adults were collected from rat (definitive host) brain and lung at day 21 (L3), 28 (L4) and 48 days (adult) post-infection of *A. cantonensis* L3s. Total RNAs were extracted from the different stages of worms using Trizol reagent. mRNA samples in equivalent amounts were reverse-transcribed into cDNA using the Revert Aid First Strand cDNA Synthesis Kit (Invitrogen Corporation, Carlsbad, CA). Real-time PCR was performed with SYBR Premix Ex Taq II (Takara, Shiga, Japan) in a LightCycler 480 Real-time PCR System (Roche, Mannheim, Germany) in accordance with the manufacturer’s protocol. β-actin was used as the internal control. The specific primer sets are listed in **Table S8**. Transcription levels were normalized to that of the housekeeping gene (β-actin) using the 2^−ΔΔCt^ method. To determine consistency between RNA-seq and qPCR, we used normalized RPKM for RNA-seq and fold-change for qPCR. The expression of genes in six samples was normalized 0~100 by setting the maximum to 100. Pheatmap (https://cran.r-project.org/ web/packages/pheatmap) in the R package was used for visualization (**Figures S10** and **S18**). Pearson and Spearman correlation analyses were performed using R [38].

**4. Genome evolution**

**4.1 Gene family analysis**

Whole-genome proteins of *C. elegans*, *A. suum*, *T. spiralis*, *B. malayi*, *M. hapla*, *N. americanus* and *H. contortus* were downloaded from Wormbase (WS246). If a gene had more than one transcript, the longest transcript was used. We used the OrthoMCL pipeline[39] to identify gene clusters/families among *A. cantonensis*, *C. elegans*, *Ascaris suum*, *Trichinella spiralis*, *Brugia malayi*, *Meloidogyne hapla*, *N. americanus* and *H. contortus*. The species-specific gene families were determined according to the presence or absence of genes for a given species.

A total of 8,723 gene clusters (families) were identified in *A. cantonensis.* We found that 2,370 gene families were shared by all eight nematodes in which 788 contained only one copy in each species and were regarded as one-to-one orthologous genes. In addition, 159 families were ʺuniqueʺ to *A. cantonensis* (**Table S5**)*.* GO enrichment analysis (see 3) indicated that a significant portion of these candidate genes were associated with ʺsuperoxide dismutase activityʺ and ʺmetallopeptidase activityʺ (**Figure S4**).

**4.2 Phylogenetic analysis**

We obtained 788 one-to-one orthologous genes among eight nematode species using the pipeline described in the section 4.1. The nucleotide sequences of these genes were aligned using MUSCLE (v3.8.31) with the default parameters [40]. Then poorly aligned regions were removed using trimAl [41] (v1.2) with the parameter “-gt 0.5”. RAxML (v8.2) was used to estimate individual gene tree under GTRGAMMA model. Finally, ASTRAL [42] (v5.6.1) was used to build species tree (**Figure S5**).

Trematoda, a class within the phylum Platyhelminthes, such as *Schistosoma japonicum, S. mansoni, S. haematobium, Opisthorchis viverrini and Clonorchis sinensis,* like *A. cantonensis,* also utilize snails as intermediate hosts. A comparison between species tree and gene tree will provide signal of potentially convergent evolution between nematodes and flukes[43]. whole-genome protein sequences of a free-living Platyhelminthes , *Schmidtea mediterranea* [44]*,* and five flukes (*S. japonicum*, *S. mansoni*, *S. haematobium*, *O. viverrini* and *C. sinensis*) were downloaded from WormBase Parasite database [45] (version WBPS5). Using the gene family analysis pipeline described in the **section 4.1**, we identified 173 one-to-one orthologous genes among the 14 species (8 nematodes and 6 flatworms). The phylogeny was built using above method (Fig 3a in the main text).

The topological structure of Platyhelminthes branch is the same as reported by Neil D Young [46].

**4.3 Divergence time inference and gene family evolution**

We estimated divergence times by PAML MCMCTREE [47] (v4.5). The Markov chain Monte Carlo (MCMC) process was run for 200,000 iterations with a sample frequency of 500 after a burn-in of 20,000 iterations, and other parameters were set defaults. Two independent runs were performed to check convergence. The following constraints were used for time calibrations (<http://www.timetree.org/>) [48]: (i) the *T. spiralis* and *C. elegans* divergence time (~428 mya); (ii) the *B. malayi* and *C. elegans* divergence time (~241 mya).

Using the gene family results and the phylogenetic tree of the eight [nematode](javascript:void(0);)s in **4.2** as inputs, we studied the expansion and contraction of gene families using CAFE (Computational Analysis of gene Family Evolution, v2.1) [49], which infers the dynamics of the gene family under a stochastic birth and death model. The CAFE identified 145 gene families with size a significantly changed size for the *A. cantonensis* lineage (Viterbi *P*<=0.05). 26 and 119 gene families were statically expanded and contracted in the *A. cantonensis* genome (**Figure S6**, **Tables S6** and **S7).**

1. **Identification, evolution and expression of specific gene or gene families**

The associations between the gene ID named in this study and the corresponding gene ID in the public database (Wormbase) are listed in **Table S9**.

Based on the gene family analyses, we have identified some expanded or specific genes in *A. cantonensis* that might be related to parasitism or adaption. To further investigate the evolution of these genes, we performed a comparison with that of other species. To avoid bias induced by different gene annotation pipelines, different software parameters, or different homologous sequences, among others, in those draft genomes, we employed a uniform annotation of these genes or gene families based on a homology-based approach in the genome of the compared species. For other species, we also manually checked these reannotated genes against their whole-genome genes. The identification and evolution of each gene families are detailed described in the following section.

**5.1 Identification and evolution of the superoxide dismutase (SOD) gene family in eight nematodes, five snail-borne flukes and the free-living platyhelminth, *Schmidtea mediterranea***

Superoxide dismutase (SODs), is an enzyme that catalyses the decomposition of superoxide, and are the first reactive species in the reduction of molecular oxygen into hydrogen peroxide and molecular oxygen[50]. There are three isozymes of the SOD gene family in mammals, namely, SOD1 (cytoplasm), SOD2 (mitochondria) and SOD3 (extracellular, EC-SOD). This enzyme has been characterized and cloned from various helminths, and SODs are postulated to play a role in the protection of parasites against the cellular, oxygen-mediated killing mechanisms by hosts [51].

SOD family protein-coding sequences of nematodes deposited in the Swiss-Prot database (release 2015-05) were retrieved. Then we performed homology-based prediction using the genomes of 8 nematodes, five flukes and *S. mediterranea* with the method described in section 2.1, except with a threshold alignment rate of > 50% and identity > 30%. The alignment rate > 50% aimed to avoid the incorrect prediction of one gene into multi-fragments incorrectly. To control false positives, genes without the “superoxide dismutase” domain (PF00080, PF00081 and [PF02777](http://pfam.sanger.ac.uk/family?acc=PF02777)) were discarded. Furthermore, candidate potential pseudogenes containing premature termination codon or frame shift mutations (1-8 bp but not 3 or 6 bp indel) in the predicted CDS were also discarded. The final SOD members of each species are shown in **Figure S7**. The phylogeny was constructed as using the following steps. a) We aligned the protein sequences of all SOD genes using MUSCLE software. b) we filtered the poorly aligned regions using trimAI [41] (v1.2) with the parameters “-gt 0.4”. c) We used the PhyML [52] (v3.0) software with the model of “WAG+GAMMA”, which was estimated as the best model by ProtTest [53] (v3.4.2), to construct a phylogenetic tree based on the protein sequences alignment.

To further confirm the topology of the “GBH” clade, we used a different software and algorithm (IQ-TREE [54] (v1.6.5), RAxML [55] (v8.2) and MrBayes [56] (v3.1.2)) to construct the phylogeny of CuZnSOD based on the “Sod_Cu” domain with the “WAG” substitution amino acid model. The topologies generated using these three programs were almost identical with reliable support values for “GBH” clades (**Figure S8**). This result supports the “GBH” branch shown in Fig 3b in the main text.

To verify these ten copies reliability at the genomic level, we conducted polymerase chain reaction (PCR) amplification and Sanger sequencing. One sequence-specific forward and reverse primer were designed (**Table S8**). The PCR products confirmed the presence of ten copies (**Figure S9**) and sequencing of the PCR products revealed high identity (0.93~1.0) compared with our predicted genomic sequences of SOD3 by BLASTN.

To further confirm the potential functional EC-SOD, we selected P51547-D2 for the following experiments in mRNA, protein and enzyme activity detection experiments.

**Quantitative real-time polymerase chain reaction (qPCR) analysis of the gene expression of P51547-D2**. L1s, L3s were collected from rat feces, digested tissue of *B. glabrata*. The larvae collected from rat (definitive host) and mouse (incidental host) brain at day 21 and 28 post-infection of *A. cantonensis*. Total RNAs were extracted from the different stages of worms using Trizol reagent. mRNA samples in equivalent amounts were reverse-transcribed into cDNA using the Revert Aid First Strand cDNA Synthesis Kit (Invitrogen Corporation, Carlsbad, CA). Real-time PCR was performed with SYBR Premix Ex Taq II (Takara, Shiga, Japan) in a LightCycler 480 Real-time PCR System (Roche, Mannheim, Germany) in accordance with the manufacturer’s protocol. The specific primer sets were as follows (5’ to 3’): P51547-D2, forward GATTAACGGCAGCGTATCT, reverse CGGTGTGATGAGGTTTCCA; β-actin, forward GGCATCCTGACCCTGAAGTA, reverse CTCTCAGCTGTGGTGGTGAA. Transcript levels were normalized to that of the housekeeping gene β-actin using the 2−ΔΔCt method as previously described (**Figure S10)**. We observed the expression of P51547-D2 in the different developmental stages residing in snail and rat. Interestingly, we found significant differential expression patterns of P51547-D2 upon *A. cantonensis* infection in mice and rats.

**Gene cloning, Expression, Purification and Determination of P51547-D2 activity.** ORF of SOD3 from cDNA was subcloned with following primer sets as followed: SOD3, forward CGCCCGGGTCGATGATGTTGCTACGTGC, reverse CGCTCGAGTTAGACGATCTCGATGATTCC. PCR products were analysed on 1% agarose gels, purified from the gel, and cloned into the pGEX-4T-1 vector with the glutathione S-transferase (GST) tag (Takara, Shiga, Japan). Each ligation was transformed into Escherichia coli BL21 competent cells (Invitrogen Corporation, Carlsbad, CA). The BL21 cells containing the recombinant plasmids were grown and expressed the enzyme in LB liquid medium at 37°C with shaking at 200 rpm for 6h.

The soluble P51547-D2 protein was purified using the GSTrap FFresin column (Amersham Pharmacia, USA). The enzyme activity of recombinant P51547-D2 was detected using the SOD Determination Kit (Sigma–Aldrich, St. Louis, MO). The absorbance was measured at 450 nm. SOD Standard Solution was prepared with dilution buffer as follows: 200 U/ml, 100 U/ml, 50 U/ml, 20 U/ml, 10 U/ml, 5 U/ml, 1 U/ml, 0.1 U/ml, 0.05 U/ml, 0.01 U/ml, and 0.001 U/ml to generate a standard inhibition curve to calculate the activities (U/ml) of P51547-D2 (**Figure S11**)

The insoluble cell fractions were separated by centrifugation and incubated in the lysis buffer (50 mM Tris/HCl, 100 mM NaCl, 6 M urea, 1% Triton X-100, pH 8.0) for 30 min, and centrifuged at 12000rpm for 10 min at 4°C. P51547-D2-GST was separated by SDS-PAGE and the gel was ground to a powder and mixed with Freund’s adjuvant (first immunization, Sigma) and incomplete Freund’s adjuvant (boosting immunizations) to prepare the P51547-D2 antibody (**Figure S11**).

- 1. **Identification and evolution of three gene families of protease related to the degradation of host hemoglobin or nutrient uptake among eight nematodes**

In addition, we observed peptidases, such as legumain (*Lgmn*), and Neprilysin-1 (NEP-1) and Aspartic protease 6 (*Asp-6*) present in the *A. cantonensis* specific or expanded gene family. These peptidases are associated with the degradation of host hemoglobin (Hb) in blood-meal parasites, such as *H. contortus* and hookworms [57-59].

The *Asp-6*, NEP-1 and *Lgmn* belong to the A01, M13 and C13 family according to the MEROPS database [60], a peptidase database. We downloaded all the protein sequences of A01, C01, M13 and C13 belonging to nematodes from the MEROPS database (download in Nov-2016). We performed the homology-based prediction as described above in the 8 nematodes. Because of the protein sequences from the MEROPS database are mainly pepunits (peptidase units and inhibitor units of all the peptidases and peptidase inhibitors), and not the full-length of genes. So, we adopted a stricter threshold, 70% alignment rate. We also only retained the genes containing the ʺEukaryotic aspartyl proteaseʺ, “Peptidase_C1”, ʺPeptidase family M13ʺ and ʺPeptidase C13 familyʺ domains (PF00026, PF00112, PF01431 and PF01650 respectively). The phylogenetic analysis was performed with the pipeline ʺMUSCLE+TrimAl+ProtTest+Phymlʺ as in the SOD family analysis. The trees were shown in **Figures S13-16**.

- 1. **Identification and evolution of the astacin-like gene family evolution in eight nematodes**

Astacin-like genes are a large family of zinc metalloproteases that are found in bacteria and animals. They have diverse roles ranging from the digestion of food to the processing of extracellular matrix components. A large number of astacin-like genes have been identified in the genome of *C. elegans* and other parasitic nematodes, such as *Strongyloides ratti*, *H. contortus*, and *N. americanus* [61, 62].

To investigate astacin-like gene family evolution on a genome-wide level, we downloaded the astacin-like protein sequences of *C. elegans* from the Swiss-Prot database (release 2015-05). The astacin-like family has more members than the SOD family (40 astacin-like genes compared with 5 SOD genes in *C. elegans,* respectively in the Swiss-Prot database) and the sequences of the astacin-like family are poorly conserved. Genes that expanded after species differentiation may not be predicted because of the poor conservation and the distant relationships among the eight nematodes. Thus, after 1-round prediction, we merged the astacin-like genes from the Swiss-Prot database and the 1-round prediction genes in eight nematodes to act as a new database and performed 2-round prediction. To avoid false positives, genes without the ASTACIN domain (IPR001506) were discarded. Candidate pseudogenes and short genes less than 200 AAs (The length of the astacin domain is of approximately 200 aa [63]) were also discarded. The final members of the Astacin-like family were annotated against the MEROPS database [64] using BLAST. Finally, we performed a protein multi-global alignment for all ASTACIN genes from eight nematodes using MUSCLE software and filtered the poorly aligned regions using trimAI with the parameters ʺ-gt 0.6ʺ. The best model (ʺVT+Gʺ) for the ML method was estimated using ProtTest. The PhyML software [52] was used to construct a phylogenetic tree with the model of ʺVT + gammaʺ. The phylogenetic tree (**Fig. 5a**) showed that MTP-1 underwent two expansion in the *A. cantonensis* genome.Recently, the astacin-like gene family was also reported to have expanded in *S. ratti* (Clade IV)*.* To illustrate the relationship of the expanded members of the astacin-like gene family across different nematodes, we used the same pipeline for re-annotated astacin-like encoding genes in *S. ratti* and conducted a phylogenetic analysis (**Figure S20**). The complex evolutionary of astacins in nematodes, especially, parasitic nematodes, may be related to its diverse functions and parasitism.

**References**

1. Wang QP, Lai DH, Zhu XQ, Chen XG, Lun ZR. Human angiostrongyliasis. Lancet Infect Dis. 2008;8(10):621-30. Epub 2008/10/17. doi: 10.1016/S1473-3099(08)70229-9. PubMed PMID: 18922484.

2. Barratt J, Chan D, Sandaradura I, Malik R, Spielman D, Lee R, et al. *Angiostrongylus cantonensis*: a review of its distribution, molecular biology and clinical significance as a human pathogen. Parasitology. 2016;143(9):1087-118. Epub 2016/05/27. doi: 10.1017/S0031182016000652. PubMed PMID: 27225800.

3. Wang QP, Wu ZD, Wei J, Owen RL, Lun ZR. Human *Angiostrongylus cantonensis*: an update. Eur J Clin Microbiol Infect Dis. 2012;31(4):389-95. Epub 2011/07/05. doi: 10.1007/s10096-011-1328-5. PubMed PMID: 21725905.

4. Dard C, Piloquet JE, Qvarnstrom Y, Fox LM, M'Kada H, Hebert JC, et al. First Evidence of Angiostrongyliasis Caused by *Angiostrongylus cantonensis* in Guadeloupe, Lesser Antilles. Am J Trop Med Hyg. 2017;96(3):692-7. Epub 2017/01/11. doi: 10.4269/ajtmh.16-0792. PubMed PMID: 28070007; PubMed Central PMCID: PMCPMC5361547.

5. Fellner A, Hellmann MA, Kolianov V, Bishara J. A non-travel related case of *Angiostrongylus cantonensis* eosinophilic meningomyelitis acquired in Israel. J Neurol Sci. 2016;370:241-3. Epub 2016/10/25. doi: 10.1016/j.jns.2016.09.058. PubMed PMID: 27772767.

6. Lammers AJ, Goorhuis A, van de Beek D, Grobusch MP, Bart A, van Gool T, et al. Eosinophilia a deux: a brain nagging souvenir from the Philippines. Infection. 2015;43(5):615-7. Epub 2015/05/07. doi: 10.1007/s15010-015-0785-7. PubMed PMID: 25944569.

7. Nguyen Y, Rossi B, Argy N, Baker C, Nickel B, Marti H, et al. Autochthonous Case of Eosinophilic Meningitis Caused by *Angiostrongylus cantonensis*, France, 2016. Emerg Infect Dis. 2017;23(6):1045-6. Epub 2017/05/19. doi: 10.3201/eid2306.161999. PubMed PMID: 28518042; PubMed Central PMCID: PMCPMC5443449.

8. Peng Y, Liu X, Pan S, Xie Z, Wang H. Anti-N-methyl-D-aspartate receptor encephalitis associated with intracranial *Angiostrongylus cantonensis* infection: a case report. Neurol Sci. 2017;38(4):703-6. Epub 2016/10/26. doi: 10.1007/s10072-016-2718-3. PubMed PMID: 27778112.

9. Qvarnstrom Y, Xayavong M, da Silva AC, Park SY, Whelen AC, Calimlim PS, et al. Real-Time Polymerase Chain Reaction Detection of *Angiostrongylus cantonensis* DNA in Cerebrospinal Fluid from Patients with Eosinophilic Meningitis. Am J Trop Med Hyg. 2016;94(1):176-81. Epub 2015/11/04. doi: 10.4269/ajtmh.15-0146. PubMed PMID: 26526920; PubMed Central PMCID: PMCPMC4710426.

10. Ueda M, Takeuchi Y, Ochiai J, Mabuchi C, Niwa J. [A case of myelitis with eosinophilia of the cerebrospinal fluid]. Rinsho Shinkeigaku. 2015;55(9):651-3. Epub 2015/07/15. doi: 10.5692/clinicalneurol.cn-000698. PubMed PMID: 26165808.

11. Vazquez JJ, Boils PL, Sola JJ, Carbonell F, de Juan Burgueno M, Giner V, et al. Angiostrongyliasis in a European patient: a rare cause of gangrenous ischemic enterocolitis. Gastroenterology. 1993;105(5):1544-9. Epub 1993/11/01. doi: 10.1016/0016-5085(93)90163-7. PubMed PMID: 8224660.

12. Kim JR, Hayes KA, Yeung NW, Cowie RH. Diverse gastropod hosts of *Angiostrongylus cantonensis*, the rat lungworm, globally and with a focus on the Hawaiian Islands. PLoS One. 2014;9(5):e94969. Epub 2014/05/03. doi: 10.1371/journal.pone.0094969. PubMed PMID: 24788772; PubMed Central PMCID: PMCPMC4008484.

13. Vurture GW, Sedlazeck FJ, Nattestad M, Underwood CJ, Fang H, Gurtowski J, et al. GenomeScope: fast reference-free genome profiling from short reads. Bioinformatics. 2017;33(14):2202-4. Epub 2017/04/04. doi: 10.1093/bioinformatics/btx153. PubMed PMID: 28369201; PubMed Central PMCID: PMCPMC5870704.

14. Chin CS, Peluso P, Sedlazeck FJ, Nattestad M, Concepcion GT, Clum A, et al. Phased diploid genome assembly with single-molecule real-time sequencing. Nat Methods. 2016;13(12):1050-4. Epub 2016/11/01. doi: 10.1038/nmeth.4035. PubMed PMID: 27749838; PubMed Central PMCID: PMCPMC5503144.

15. Li R, Fan W, Tian G, Zhu H, He L, Cai J, et al. The sequence and de novo assembly of the giant panda genome. Nature. 2010;463(7279):311-7. Epub 2009/12/17. doi: 10.1038/nature08696. PubMed PMID: 20010809; PubMed Central PMCID: PMCPMC3951497.

16. Marcais G, Kingsford C. A fast, lock-free approach for efficient parallel counting of occurrences of k-mers. Bioinformatics. 2011;27(6):764-70. Epub 2011/01/11. doi: 10.1093/bioinformatics/btr011. PubMed PMID: 21217122; PubMed Central PMCID: PMCPMC3051319.

17. Daccord N, Celton JM, Linsmith G, Becker C, Choisne N, Schijlen E, et al. High-quality de novo assembly of the apple genome and methylome dynamics of early fruit development. Nat Genet. 2017;49(7):1099-106. Epub 2017/06/06. doi: 10.1038/ng.3886. PubMed PMID: 28581499.

18. Kajitani R, Toshimoto K, Noguchi H, Toyoda A, Ogura Y, Okuno M, et al. Efficient de novo assembly of highly heterozygous genomes from whole-genome shotgun short reads. Genome Res. 2014;24(8):1384-95. Epub 2014/04/24. doi: 10.1101/gr.170720.113. PubMed PMID: 24755901; PubMed Central PMCID: PMCPMC4120091.

19. Ye C, Hill CM, Wu S, Ruan J, Ma ZS. DBG2OLC: Efficient Assembly of Large Genomes Using Long Erroneous Reads of the Third Generation Sequencing Technologies. Sci Rep. 2016;6:31900. Epub 2016/08/31. doi: 10.1038/srep31900. PubMed PMID: 27573208; PubMed Central PMCID: PMCPMC5004134.

20. Walker BJ, Abeel T, Shea T, Priest M, Abouelliel A, Sakthikumar S, et al. Pilon: an integrated tool for comprehensive microbial variant detection and genome assembly improvement. PLoS One. 2014;9(11):e112963. Epub 2014/11/20. doi: 10.1371/journal.pone.0112963. PubMed PMID: 25409509; PubMed Central PMCID: PMCPMC4237348.

21. Boetzer M, Pirovano W. SSPACE-LongRead: scaffolding bacterial draft genomes using long read sequence information. BMC Bioinformatics. 2014;15:211. Epub 2014/06/22. doi: 10.1186/1471-2105-15-211. PubMed PMID: 24950923; PubMed Central PMCID: PMCPMC4076250.

22. Boetzer M, Henkel CV, Jansen HJ, Butler D, Pirovano W. Scaffolding pre-assembled contigs using SSPACE. Bioinformatics. 2011;27(4):578-9. Epub 2010/12/15. doi: 10.1093/bioinformatics/btq683. PubMed PMID: 21149342.

23. Parra G, Bradnam K, Korf I. CEGMA: a pipeline to accurately annotate core genes in eukaryotic genomes. Bioinformatics. 2007;23(9):1061-7. Epub 2007/03/03. doi: 10.1093/bioinformatics/btm071. PubMed PMID: 17332020.

24. Simao FA, Waterhouse RM, Ioannidis P, Kriventseva EV, Zdobnov EM. BUSCO: assessing genome assembly and annotation completeness with single-copy orthologs. Bioinformatics. 2015;31(19):3210-2. Epub 2015/06/11. doi: 10.1093/bioinformatics/btv351. PubMed PMID: 26059717.

25. Grabherr MG, Haas BJ, Yassour M, Levin JZ, Thompson DA, Amit I, et al. Full-length transcriptome assembly from RNA-Seq data without a reference genome. Nat Biotechnol. 2011;29(7):644-52. Epub 2011/05/17. doi: 10.1038/nbt.1883. PubMed PMID: 21572440; PubMed Central PMCID: PMCPMC3571712.

26. Birney E, Clamp M, Durbin R. GeneWise and Genomewise. Genome Res. 2004;14(5):988-95. Epub 2004/05/05. doi: 10.1101/gr.1865504. PubMed PMID: 15123596; PubMed Central PMCID: PMCPMC479130.

27. Trapnell C, Pachter L, Salzberg SL. TopHat: discovering splice junctions with RNA-Seq. Bioinformatics. 2009;25(9):1105-11.

28. Trapnell C, Roberts A, Goff L, Pertea G, Kim D, Kelley DR, et al. Differential gene and transcript expression analysis of RNA-seq experiments with TopHat and Cufflinks. Nature protocols. 2012;7(3):562-78.

29. Bairoch A, Apweiler R. The SWISS-PROT protein sequence database and its supplement TrEMBL in 2000. Nucleic Acids Res. 2000;28(1):45-8. PubMed PMID: 10592178; PubMed Central PMCID: PMCPMC102476.

30. Ogata H, Goto S, Sato K, Fujibuchi W, Bono H, Kanehisa M. KEGG: Kyoto Encyclopedia of Genes and Genomes. Nucleic Acids Res. 1999;27(1):29-34. PubMed PMID: 9847135; PubMed Central PMCID: PMCPMC148090.

31. Zdobnov EM, Apweiler R. InterProScan--an integration platform for the signature-recognition methods in InterPro. Bioinformatics. 2001;17(9):847-8. PubMed PMID: 11590104.

32. Tarailo-Graovac M, Chen N. Using RepeatMasker to identify repetitive elements in genomic sequences. Curr Protoc Bioinformatics. 2009;Chapter 4:Unit 4 10. doi: 10.1002/0471250953.bi0410s25. PubMed PMID: 19274634.

33. Jurka J, Kapitonov VV, Pavlicek A, Klonowski P, Kohany O, Walichiewicz JJC, et al. Repbase Update, a database of eukaryotic repetitive elements. 2005;110(1-4):462-7.

34. Edgar RC, Myers EW. PILER: identification and classification of genomic repeats. Bioinformatics. 2005;21 Suppl 1:i152-8. Epub 2005/06/18. doi: 10.1093/bioinformatics/bti1003. PubMed PMID: 15961452.

35. Price AL, Jones NC, Pevzner PA. De novo identification of repeat families in large genomes. Bioinformatics. 2005;21 Suppl 1:i351-8. doi: 10.1093/bioinformatics/bti1018. PubMed PMID: 15961478.

36. Xu Z, Wang H. LTR_FINDER: an efficient tool for the prediction of full-length LTR retrotransposons. Nucleic Acids Res. 2007;35(Web Server issue):W265-8. doi: 10.1093/nar/gkm286. PubMed PMID: 17485477; PubMed Central PMCID: PMCPMC1933203.

37. !!! INVALID CITATION !!! 43.

38. R Development Core Team RFFSC. R: A Language and Environment for Statistical Computing. Vienna Austria R Foundation for Statistical Computing. 2008;1:ISBN 3-900051-07-0. doi: 10.1007/978-3-540-74686-7. PubMed PMID: 1505.

39. Li L, Stoeckert CJ, Jr., Roos DS. OrthoMCL: identification of ortholog groups for eukaryotic genomes. Genome Res. 2003;13(9):2178-89. doi: 10.1101/gr.1224503. PubMed PMID: 12952885; PubMed Central PMCID: PMCPMC403725.

40. Edgar RC. MUSCLE: multiple sequence alignment with high accuracy and high throughput. Nucleic Acids Research. 2004;32(5):1792-7. doi: Doi 10.1093/Nar/Gkh340. PubMed PMID: ISI:000220487200025.

41. Capella-Gutierrez S, Silla-Martinez JM, Gabaldon T. trimAl: a tool for automated alignment trimming in large-scale phylogenetic analyses. Bioinformatics. 2009;25(15):1972-3. Epub 2009/06/10. doi: 10.1093/bioinformatics/btp348. PubMed PMID: 19505945; PubMed Central PMCID: PMCPMC2712344.

42. Mirarab S, Reaz R, Bayzid MS, Zimmermann T, Swenson MS, Warnow T. ASTRAL: genome-scale coalescent-based species tree estimation. Bioinformatics. 2014;30(17):i541-8. Epub 2014/08/28. doi: 10.1093/bioinformatics/btu462. PubMed PMID: 25161245; PubMed Central PMCID: PMCPMC4147915.

43. Li Y, Liu Z, Shi P, Zhang J. The hearing gene Prestin unites echolocating bats and whales. Curr Biol. 2010;20(2):R55-6. doi: 10.1016/j.cub.2009.11.042. PubMed PMID: 20129037.

44. Robb SM, Gotting K, Ross E, Sanchez Alvarado A. SmedGD 2.0: The Schmidtea mediterranea genome database. Genesis. 2015;53(8):535-46. Epub 2015/07/04. doi: 10.1002/dvg.22872. PubMed PMID: 26138588; PubMed Central PMCID: PMCPMC4867232.

45. Howe KL, Bolt BJ, Shafie M, Kersey P, Berriman M. WormBase ParaSite - a comprehensive resource for helminth genomics. Mol Biochem Parasitol. 2017;215:2-10. Epub 2016/12/03. doi: 10.1016/j.molbiopara.2016.11.005. PubMed PMID: 27899279; PubMed Central PMCID: PMCPMC5486357.

46. !!! INVALID CITATION !!! [48].

47. Yang Z. PAML 4: phylogenetic analysis by maximum likelihood. Mol Biol Evol. 2007;24(8):1586-91. Epub 2007/05/08. doi: 10.1093/molbev/msm088. PubMed PMID: 17483113.

48. Rota-Stabelli O, Daley AC, Pisani D. Molecular timetrees reveal a Cambrian colonization of land and a new scenario for ecdysozoan evolution. Curr Biol. 2013;23(5):392-8. Epub 2013/02/05. doi: 10.1016/j.cub.2013.01.026. PubMed PMID: 23375891.

49. De Bie T, Cristianini N, Demuth JP, Hahn MW. CAFE: a computational tool for the study of gene family evolution. Bioinformatics. 2006;22(10):1269-71. Epub 2006/03/18. doi: 10.1093/bioinformatics/btl097. PubMed PMID: 16543274.

50. Kim TS, Jung Y, Na BK, Kim KS, Chung PR. Molecular cloning and expression of Cu/Zn-containing superoxide dismutase from *Fasciola hepatica*. Infect Immun. 2000;68(7):3941-8. Epub 2000/06/17. doi: 10.1128/iai.68.7.3941-3948.2000. PubMed PMID: 10858207; PubMed Central PMCID: PMCPMC101671.

51. Callahan HL, Crouch RK, James ER. *Dirofilaria immitis* superoxide dismutase: purification and characterization. Mol Biochem Parasitol. 1991;49(2):245-51. Epub 1991/12/01. doi: 10.1016/0166-6851(91)90068-h. PubMed PMID: 1775168.

52. Guindon S, Delsuc F, Dufayard JF, Gascuel O. Estimating maximum likelihood phylogenies with PhyML. Methods Mol Biol. 2009;537:113-37. Epub 2009/04/21. doi: 10.1007/978-1-59745-251-9_6. PubMed PMID: 19378142.

53. Abascal F, Zardoya R, Posada D. ProtTest: selection of best-fit models of protein evolution. Bioinformatics. 2005;21(9):2104-5. Epub 2005/01/14. doi: 10.1093/bioinformatics/bti263. PubMed PMID: 15647292.

54. Nguyen LT, Schmidt HA, von Haeseler A, Minh BQ. IQ-TREE: a fast and effective stochastic algorithm for estimating maximum-likelihood phylogenies. Mol Biol Evol. 2015;32(1):268-74. Epub 2014/11/06. doi: 10.1093/molbev/msu300. PubMed PMID: 25371430; PubMed Central PMCID: PMCPMC4271533.

55. Stamatakis A. RAxML version 8: a tool for phylogenetic analysis and post-analysis of large phylogenies. Bioinformatics. 2014;30(9):1312-3. Epub 2014/01/24. doi: 10.1093/bioinformatics/btu033. PubMed PMID: 24451623; PubMed Central PMCID: PMCPMC3998144.

56. Ronquist F, Teslenko M, van der Mark P, Ayres DL, Darling A, Hohna S, et al. MrBayes 3.2: efficient Bayesian phylogenetic inference and model choice across a large model space. Syst Biol. 2012;61(3):539-42. Epub 2012/02/24. doi: 10.1093/sysbio/sys029. PubMed PMID: 22357727; PubMed Central PMCID: PMCPMC3329765.

57. Williamson AL, Brindley PJ, Knox DP, Hotez PJ, Loukas A. Digestive proteases of blood-feeding nematodes. Trends Parasitol. 2003;19(9):417-23. Epub 2003/09/06. PubMed PMID: 12957519.

58. Dalton JP, Brindley PJ, Donnelly S, Robinson MW. The enigmatic asparaginyl endopeptidase of helminth parasites. Trends Parasitol. 2009;25(2):59-61. Epub 2008/12/23. doi: 10.1016/j.pt.2008.11.002. PubMed PMID: 19101207.

59. Ni F, Wang Y, Zhang J, Yu L, Fang W, Luo D. Cathepsin B-like and hemoglobin-type cysteine proteases: stage-specific gene expression in *Angiostrongy cantonensis*. Exp Parasitol. 2012;131(4):433-41. Epub 2012/06/07. doi: 10.1016/j.exppara.2012.05.014. PubMed PMID: 22668746.

60. Rawlings ND, Barrett AJ, Finn R. Twenty years of the MEROPS database of proteolytic enzymes, their substrates and inhibitors. Nucleic Acids Res. 2016;44(D1):D343-50. Epub 2015/11/04. doi: 10.1093/nar/gkv1118. PubMed PMID: 26527717; PubMed Central PMCID: PMCPMC4702814.

61. Park JO, Pan J, Mohrlen F, Schupp MO, Johnsen R, Baillie DL, et al. Characterization of the astacin family of metalloproteases in C. elegans. BMC Dev Biol. 2010;10:14. Epub 2010/01/30. doi: 10.1186/1471-213X-10-14. PubMed PMID: 20109220; PubMed Central PMCID: PMCPMC2824743.

62. Hunt VL, Tsai IJ, Coghlan A, Reid AJ, Holroyd N, Foth BJ, et al. The genomic basis of parasitism in the *Strongyloides* clade of nematodes. Nat Genet. 2016;48(3):299-307. Epub 2016/02/02. doi: 10.1038/ng.3495. PubMed PMID: 26829753; PubMed Central PMCID: PMCPMC4948059.

63. Möhrlen F, Hutter H, Zwilling R. The astacin protein family in *Caenorhabditis elegans*. The FEBS Journal. 2003;270(24):4909-20.

64. Rawlings ND, Morton FR, Kok CY, Kong J, Barrett AJ. MEROPS: the peptidase database. Nucleic Acids Res. 2008;36(Database issue):D320-5. doi: 10.1093/nar/gkm954. PubMed PMID: 17991683; PubMed Central PMCID: PMCPMC2238837.
